# PIP-SNP: a pipeline for processing SNP data featured as linkage disequilibrium bin mapping, genotype imputing and marker synthesizing

**DOI:** 10.1093/nargab/lqab060

**Published:** 2021-07-05

**Authors:** Wenchao Zhang, Yun Kang, Xinbin Dai, Shizhong Xu, Patrick X Zhao

**Affiliations:** Noble Research Institute LLC, 2510 Sam Noble Parkway, Ardmore, OK 73401, USA; Noble Research Institute LLC, 2510 Sam Noble Parkway, Ardmore, OK 73401, USA; Noble Research Institute LLC, 2510 Sam Noble Parkway, Ardmore, OK 73401, USA; Department of Botany and Plant Sciences, University of California, Riverside, CA 92521, USA; Noble Research Institute LLC, 2510 Sam Noble Parkway, Ardmore, OK 73401, USA

## Abstract

Genome-wide association study data analyses often face two significant challenges: (i) high dimensionality of single-nucleotide polymorphism (SNP) genotypes and (ii) imputation of missing values. SNPs are not independent due to physical linkage and natural selection. The correlation of nearby SNPs is known as linkage disequilibrium (LD), which can be used for LD conceptual SNP bin mapping, missing genotype inferencing and SNP dimension reduction. We used a stochastic process to describe the SNP signals and proposed two types of autocorrelations to measure nearby SNPs’ information redundancy. Based on the calculated autocorrelation coefficients, we constructed LD bins. We adopted a *k*-nearest neighbors algorithm (kNN) to impute the missing genotypes. We proposed several novel methods to find the optimal synthetic marker to represent the SNP bin. We also proposed methods to evaluate the information loss or information conservation between using the original genome-wide markers and using dimension-reduced synthetic markers. Our performance assessments on the real-life SNP data from a rice recombinant inbred line (RIL) population and a rice HapMap project show that the new methods produce satisfactory results. We implemented these functional modules in C/C++ and streamlined them into a web-based pipeline named PIP-SNP (https://bioinfo.noble.org/PIP_SNP/) for processing SNP data.

## INTRODUCTION

Due to the great success in identifying causal genetic markers conferring complex traits and diseases (1), genome-wide association studies (GWAS) and quantitative trait locus (QTL) mapping recently have revolutionized the fields of quantitative genetics (2). The high abundance of single-nucleotide polymorphisms (SNPs) along the genome has made them the most promising markers for linkage and association studies for complex traits, including complex diseases (3).

It is well known that increasing marker density and sample sizes can further increase the resolution of QTL mapping (4). Next-generation sequencing (NGS) technology (5) can provide cheap, reliable and high-throughput sequencing data (6), which are needed for high GWAS accuracy and QTL mapping resolution. Current GWAS projects mostly rely on linear mixed models (LMMs) to evaluate each marker's additive effect, which are computationally more expensive than simple linear regression analyses. Therefore, GWAS analyses and statistical tests for a large number of SNPs present a great challenge in terms of computational load (7).

The K+Q LMM (8) incorporates both the cryptic kinship relatedness and population stratification structure, and has been widely used in GWAS analysis. However, detected QTLs from GWAS are often account for only a small fraction of the heritability (9), mainly due to ignorance of other effects beyond the additive effects. An important factor that accounts for the missing heritability may come from epistatic effects defined as gene-by-gene interactions (G×G) or genotype-by-environment effects denoted by G×E (10). To account for more heritability and analyze traits with complex genetic architecture, we developed a series of novel LMMs (11) and related tools that have well addressed the two typical interaction effects: G×G and G×E (12,13). Based on these LMMs and tools, 2D association studies were proposed to detect the interaction effects. However, the number of total genetic variants has increased in a quadratic scale compared with the number of genetic variants under the additive model in the conventional GWAS models (14,15). Therefore, calculations of the interaction kinship matrix and the *P* values for interaction genetic marker pairs require much higher computing capacity (12). The parallel computing deployed with thousands of CPU or GPU nodes can only linearly decrease the full computing time (16), which can easily reach a plateau, making it impracticable to handle millions of SNPs for their epistatic effects in a 2D GWAS analysis. Alternative approaches must be considered to reduce the dimension of genetic variants to an acceptable level (17).

SNPs are not independent and the correlation of nearby SNPs is called linkage disequilibrium (LD), which can be used for SNPs’ dimension reduction. LD exists because of shared ancestry resulting in haplotype patterns, a particular combination of alleles along the contemporary chromosomes (18). Studies reported in literature suggest that the whole genome can be mapped into many blocks and within each block, SNPs are highly correlated, and a ‘tag’ SNP can represent the whole block. A small number of representative SNPs are sufficient to provide information about the haplotype block structures of the whole genome (19,20). LD block mapping and haplotype pattern analysis have been successfully used to identify DNA variations that are relevant to common and complex diseases (21–23).

NGS technology for a genome sequencing project includes a top-down digestion and fragmentation of the DNA genome, base calling and alignment of short reads to a reference or bottom-up assembly of high-quality short reads into a genome (24). Therefore, NGS data are subject to high error rates due to multiple factors, including base-calling and alignment errors. Moreover, some NGS users preferred to lower costs and chose low-coverage sequencing, which consequentially increase the difficulty in alignment and decrease the accuracy in the following SNP and genotype calling (6). In the study by Nielsen *et al.* (6), about 40% of the genotypes were recorded as non-calls and reported as missing values to ensure the accuracy of SNP calling at an acceptable level. However, association mapping requires complete genotypes and phenotypes. As a result, SNP data for GWAS are subject to a high percentage of the missing values (25), although genomic SNPs are abundant. Therefore, imputations are needed to fill the missing genotypes prior to association analyses. Additionally, imputation can further improve the power of testing in the downstream GWAS analyses (26,27). In summary, GWAS technology faces two challenges: (i) high dimensionality of SNP data and (ii) missing genotypes. It is necessary to develop methods and tools to overcome these two challenges. To the best of our knowledge, there are no tools available to resolve both challenges in one-stop processing.

In this study, we developed a web-based pipeline called PIP-SNP, which has taken into account the redundant information of nearby SNPs, missing genotypes to be imputed and high-dimensional SNPs to be reduced and synthesized. We first borrowed the concepts of LD block and considered nearby SNP signals as stochastic processes, and then used the correlation and autocorrelation measurements (28) to describe the similarity of nearby SNPs. Two types of correlations have been proposed to characterize the specific haplotype patterns in a rice recombinant inbred line (RIL) population (29) and a rice HapMap population (30). First, we proposed the criteria for detecting LD conceptual bins that could partition the whole genome into LD bins. Second, we adopted a *k*-nearest neighbors method (kNN) (31), from which missing genotypes were inferred. Finally, we proposed and discussed several synthesizing methods that allowed us to find the optimal representative tag SNPs or integrative markers. Based on these proposed methods, we used C/C++ to implement each module and seamed these models as a pipeline PIP-SNP. To be more flexible, we designed the application with two distinct scenarios: to auto-detect the LD bins and to use existing LD bins, respectively. The PIP-SNP pipeline is now publicly available at https://bioinfo.noble.org/PIP_SNP/.

## MATERIALS AND METHODS

Due to linkage disequilibrium, a genome can be mapped into haplotype blocks. We can select only informative SNPs or synthetic markers representing the original block structures in the genome for genome-wide association studies (3,20). The biological block mapping should be based on evidence for historical recombination events (20), and the recombination hot spots can be defined with boundaries (32). High-density SNP markers are used to infer recombination breakpoints, which then facilitate the construction of LD bins (17).

### Stochastic processes and autocorrelation to describe nearby SNP signal

Autocorrelation is a type of serial correlation, which has been used in stochastic signal processing to measure the similarity of a signal with a delayed copy of itself as a function of delay (33). Due to LD, nearby SNPs are correlative and can be well described by a stochastic process. Let the genotyped SNPs being ordered by chromosome positions, which can be represented by an $M \times N$ matrix, where $M$ and $N$ are the SNP number and sample size, respectively. A specific SNP signal can be represented as $SN{P_i}$, which is a genotype vector with length $N$. The Pearson correlation coefficient between $SN{P_i}$ and $SN{P_j}$ is expressed by

(1) $$\begin{eqnarray*} R \left( {i, j} \right) &=& Corr \left( {SN{P_i},SN{P_j}} \right) \nonumber \\ &=& \frac{{\mathop \sum \nolimits_{n = 1}^N (SN{P_i}\left( n \right) - \overline {SN{P_i}} )(SN{P_j}\left( n \right) - \overline {SN{P_j}} )}}{{\sqrt {\mathop \sum \nolimits_{n = 1}^N {{\left( {\ SN{P_i}\left( n \right) - \overline {SN{P_i}} } \right)}^2}} \sqrt {\mathop \sum \nolimits_{n = 1}^N {{(SN{P_j}\left( n \right) - \overline {SN{P_j}} )}^2}} }} \end{eqnarray*}$$

where $i$, and $j$ are the ordered SNP indices.

A simple way to measure the relationship between two neighbor signals $SN{P_i}$ and $SN{P_{i + 1}}$ is given in Equation (2), which essentially is a specific autocorrelation and can be used to detect the boundary of a LD bin. If $SN{P_i}$ and $SN{P_{i + 1}}$ are positioned at the same haplotype block, its correlation should be high (determination of coefficient is close to 1.0); otherwise, it should be low (determination of coefficient is close to 0.0).

(2) $$\begin{equation*}NR \left( i \right) = Corr\left( {SN{P_i},SN{P_{i + 1}}} \right)\,\,i = 1,2,3,...\end{equation*}$$

Further, the similarity profile of one fix SNP signal with its continuous neighbor SNP signals is given in Equation (3), which essentially is a general autocorrelation measuring the detail on how the LD decays nearby a fixed SNP.

(3) $$\begin{equation*}{R_{{i_{ 0}}}} \left( \tau \right) = Corr\left( {SN{P_{{i_{ 0}}}},SN{P_{{i_{ 0}} + \tau }}} \right),\,\,\tau = 1,2,3,...\end{equation*}$$

### Characterization of the haplotype block pattern and detection of LD conceptual bins

**Figure 1.**
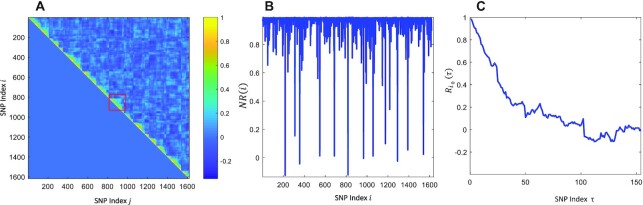
The Correlation and auto-correlation analysis of RIL rice data. (**A**) Image scale color mapping of Pearson correlation for all SNP marker pairs and LD block showing a right triangular pattern. (**B**) Line plot of the auto-correlation of neighbor two SNPs. (**C**) Line plot of auto-correlation of a fixed SNP with its right shift neighbor SNP.

**Figure 2.**
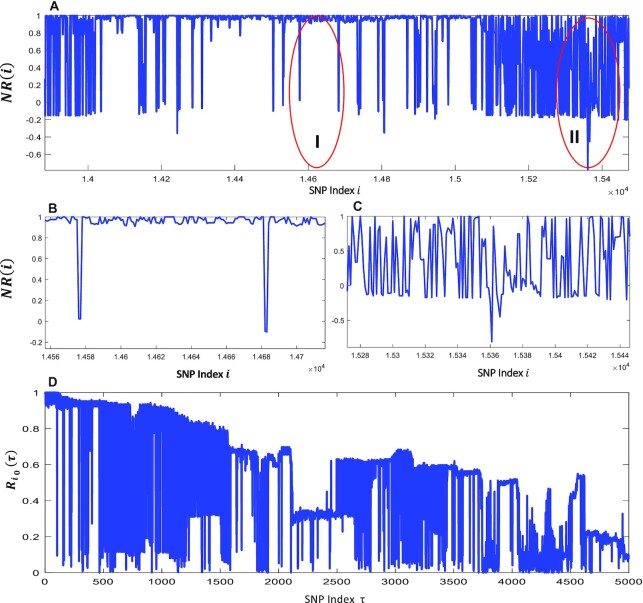
The Correlation and auto-correlation analysis of Rice HapMap data. (**A**) Line plot of the auto-correlation of neighbor two SNPs. (**B**) Zoom in the plot for smooth region I. (**C**) Zoom in the plot for spike region II. (**D**) Line plot of auto-correlation of a fixed SNP with its right shift neighbor SNP.

The correlation between two neighbor SNPs and the autocorrelation profile measuring the LD decay in a local range of the genome can be described graphically. Figures 1 and 2 give the snapshots of the above two defined autocorrelations for the SNP data from a RIL population and a HapMap population, respectively. We can find that the SNP data from the two populations show very distinct haplotype patterns and LD decay profiles.

The haplotype block pattern as LD changes is demonstrated as a right triangle in the 2D Pearson correlation of SNP pairs (Figure 1A), or a fluctuating rectangle with relatively high correlation coefficients amid a sudden drop indicating the boundaries (Figures 1B and 2A,B). To simplify, we can continuously calculate the correlation coefficient of two neighbor SNPs and compare it with a preset threshold to detect block pattern boundaries. Ideally, the boundary should correspond to a SNP at a recombination point. However, due to limited sample size and low SNP data quality during genetic variant calling, the boundary can be blurred (Figure 2A and C). In the worst-case scenario, the LD decay curve is not monotonic but contains many acute spikes (Figure 2D). As a result, we can only detect an approximate recombination point as the block boundary. Considering these, we can continuously apply the threshold to calculate the NR values (Pearson correlation between two neighboring SNPs) and the options to detect the boundary can be to consider the most right two SNPs, the most left and right SNPs and to consider both or one of the two options. In this study, once the haplotype block patterns are mapped and partitioned, we call them LD bins, which may not accurately reflect the haplotype blocks. Figure 1 is based on a typical case using RIL SNP data, which show us a monotonic LD decay and therefore is comparably easy to detect the LD conceptual bins. However, Figure 2 is based on SNP data from a diverse rice HapMap population, which demonstrated a challenging case as the non-monotonic LD decay (Figure 2C) and the worst-scenario composed with the smooth and acute spike region in the line plots of NR auto-correlation (Figure 2A–C). To detect a LD conceptual bin, we can design such an algorithm by which multiple SNPs can be clustered into a bin if their neighbor correlations $NR( i )$ are all above a preset threshold and/or the LD decay is not obviously decreased. Supplementary Note S1 and Supplementary Figure S1 provide the method details of LD bin detecting and mapping.

### The kNN algorithm and LD bin-based imputation of the missing genotypes

The NGS technology provides high-dimensional SNP markers but also suffers from more missing values. However, the downstream association analysis requires the genotype completeness for all SNPs. Therefore, imputation is a critical step in GWAS analysis, which essentially is to infer the most optimal substitute to fill the missing values. Of all the imputing methods and tools, there are two distinct categories. One is based on a phasing procedure that maps the ordered SNPs to the high quality reference genome or genotype panels, e.g. humans (34) and cattle (35). The other is a more generic method relying only on the data relatedness nearby the missing SNP values (25). In most cases, we study the non-model organisms and, unfortunately, the reference is lacking. In this case, the generic method to mine the innate correlation for imputing should be the only solution. Money D. etc. developed a tool called LinkImpute, which uses the extended kNN method to infer the missing value in a local regression region defined by the specific $k$ samples and $l$ SNPs (25). Because it requires the user to specify two fixed parameters and use the LD relatedness for imputing, the method was named as LD-kNNi.

In this study, we modified the LD-kNNi method and applied it to impute the missing values in each detected LD conceptual bins (Supplementary Note S2). For each missing genotype, the regression region was confined within its own bin and the specific $k$ ‘neighbor’ samples. Here, the $k$ ‘neighbor’ samples were selected based on the samples’ distance. Compared with the LD-kNNi method using the fix $l$ SNPs rigidly, the LD bins are concatenated with variable SNP sizes. Therefore, our method only needs to specify one parameter as $k$ samples.

### Generation of synthetic marker to represent each LD conceptual bin

A high-dimensional SNP marker can statistically improve the QTL mapping resolution, but it has reached a plateau (17) in epistatic GWAS analysis, although large-scale computational infrastructures such as parallelization of thousands of CPUs and GPUs could be deployed (12,13,16). Since the LD exists, it is reasonable to develop methods for partitioning the whole genome-wide SNPs into LD conceptual bins, and further it is possible to develop some methods to find an optimal tag SNP or generate an integrative marker to represent each detected bin.

Supposing one bin containing $b$ SNPs is represented as a $b \times N$ matrix, where the corresponding numerical genotype value is represented as $g_{i,n}$, we can use formula 4 or 5 to calculate the Euclidean norm as the integrative marker or find the optimal SNP as the tag SNP, respectively.

(4) $$\begin{equation*} {G_{bs}} = \left[ {\begin{array}{cccc} {\sqrt {\mathop \sum \nolimits_{i = 1}^b g{{_{i,1}^2}}} } & \ {\sqrt {\mathop \sum \nolimits_{i = 1}^b g_{i,2}^2} } & \ \ldots & \ {\sqrt {\mathop \sum \nolimits_{i = 1}^b g_{i,N}^2} } \end{array}} \right] \end{equation*}$$

(5) $$\begin{equation*}Tag\_SNP = \mathop {{\rm{Arg\ max }}\frac{1}{b}}\limits_{SN{P_i}} \mathop \sum \nolimits_{j = 1}^b Corr\left( {SN{P_i},\ SN{P_j}} \right) \end{equation*}$$

**Figure 3.**
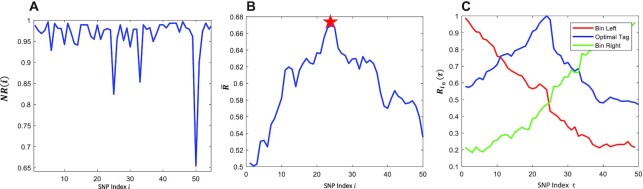
Correlation and auto-correlation of a detected LD bin and determining representative tag SNP. (**A**) Correlation analysis of neighboring two SNPs showing a LD bin boundary. (**B**) The optimal tag SNP determined by the maximal $\bar{R}$ across all SNPs in the detected LD bin. (**C**) Typical $R$ trend of the left boundary, right boundary and optimal tag SNP across all the SNPs in the bin.

Figure 3 illustrates the procedure to find the optimal SNP as the representative tag SNP in a detected LD conceptual bin. Specifically, a LD bin containing 50 SNPs will be detected if we apply 0.7 as a threshold to the calculated NR values (Figure 3A). Further, we scan all the SNPs in the bin and calculate all of the average of correlation $\overline {R{\rm{\ }}}$ for one selected SNP across all other SNPs. Finally, the SNP with the maximum $\overline {R{\rm{\ }}}$ will be selected as the optimal tag SNP to represent the detected LD conceptual bin (Figure 3B). Figure 3C highlights three specific correlation $R$ trends of the left, the right and the optimal tag SNP across the entire bin.

Regarding the binary genotype coding such as 0, 1, 2, the integrated marker will become continuous float format not following the original binary format. In addition, the synthetic marker comprehensively integrates the genetic information of the whole bin, but the resolution of the marker’s position in the chromosome will decrease into a bin. Comparably, the tag SNP still follows the same binary format and conserves the resolution of the marker’s position.

### Spike autocorrelation pattern of random SNP data and deep synthesizing

We investigated two distinct SNP data sets from a rice RIL population and a diverse rice HapMap population. We found that the autocorrelation characteristics for the two types of SNP data are quite different (Figures 1 and 2). In general, the RIL SNP data show a conservatively stable profile and modestly decreasing correlation values for the above-defined method, but the HapMap SNP data show violent vibrations and many spikes. Therefore, it is more challenging to process the random SNP data from a Hapmap population. However, the acute spike autocorrelation patterns indicate that several types of SNPs are closely entangled in a local region. The method to group and synthesize similar type of SNPs should consider not only the neighbor-joining SNPs (e.g. $SN{P_{i - 1}}$ and $SN{P_i}$) but also the neighbor-skipping SNPs (e.g. $SN{P_{i - 1}}$ and $SN{P_{i + 1}}$). Here, we developed a unique two-step method that includes an initial shallow synthesizing and an aggressive deep synthesizing (Supplementary Note S3). Supplementary Figure S2a illustrates the concept of shallow synthesizing as the first step to clump up only neighbor-joining SNPs, while the deep synthesizing as a further optional step to merge the non-adjacent SNPs. Supplementary Figure S2b shows the implementation flowchart of deep synthesizing. Deep synthesizing differs the shallow synthesizing as it considers not only the neighbor-joining SNPs but also neighbor-skipping SNPs. Compared with shallow synthesizing, the deep synthesizing can acquire a higher SNP marker reduction ratio, which can efficiently reduce the high dimensional SNP data from a HapMap population to an acceptable level and not cause too much genetic information loss.

## DESIGN AND IMPLEMENTATION

### Design overview

**Figure 4.**
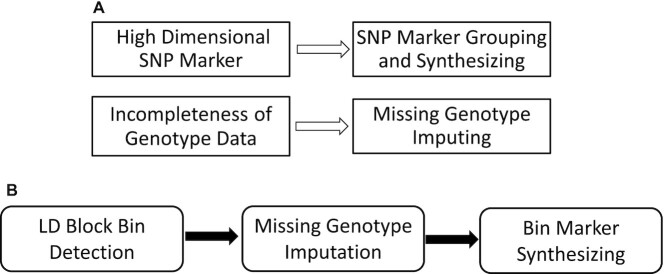
Biological concepts of pipeline PIP-SNP. (**A**) The challenges in SNP data processing. (**B**) The three main processing modules in PIP-SNP.

In general, we aimed to develop a platform to solve the two great challenges to GWAS technology: high-dimensional SNP data and the incompleteness of genotype data. Through the proposed methods rooted in the correlation analysis of SNPs, the biological Haplotype block can be mapped, the LD conceptual bins can be detected, the missing genotype values can be imputed, and the high dimensional SNP marker can be reduced to an acceptable number. Figure 4 illustrates the whole concept of the two challenges, the reasonable solutions and the main processing modules.

Most of the numerical SNP data are coded as the count of minor alleles. The biallelic SNPs have three combinational genotypes numerically as 0 (homozygous major allele), 1 (heterozygous) and 2 (homozygous minor allele), respectively. As previously mentioned, there may be many missing values that need to be imputed before conducting a GWAS analysis. To simplify, we specifically coded all the missing genotype as -1 in this study.

Along with the function implementation, ease of use also is a very important criterion. Users prefer to choose a data processing tool or platform with a friendly user interface and painless learning curve. Considering this, we developed the platform as a web pipeline, which can naturally avoid the tool’s installation and updating. Additionally, we need to consider the different scenarios in real application, which can increase the flexibility.

### Implementation of three function modules and seaming them into a web-based pipeline

Correlation analysis of the neighbor SNPs is the backbone of this study. Based on the correlation and autocorrelation analysis of one SNP and its neighbor, LD conceptual bins can be detected and the whole genome can be mapped. After finishing the LD bin mapping, the missing values in each detected bin will be imputed by the kNN method with the specific $k$ samples. Finally, one synthetic marker representing each bin either as the integration of all SNPs or a tag SNP will be generated. In general, there are three related function modules, which can be seamlessly connected and developed as a whole project. Figure 4B illustrates the diagram containing three connected modules.

Considering the high dimension of SNP variant, the computational efficiency should be seriously considered. Therefore, we chose C/C++ to implement each function module in Open-Source IDE Code:Blocks. All the source codes are compiled into executable command lines in Linux. To run this command line, users only need to provide the raw SNP data at the specific format together with the configured parameters.

Web interfaces usually can provide user-friendly convenience by maximally avoiding the mistaken parameter configuration. This motivated us to develop a web-based pipeline PIP-SNP. Generally, PIP-SNP includes a server part for the computation dense analysis and a remote-client part for a user's job submission and results downloading. We realized that the original genotyped SNP data can reach up to several GBs with millions of SNP markers and uploading such a large text file from a remote client side to the PIP-SNP’s web server can be exceedingly difficult. Therefore, we technically developed a module that can work in an HTML5 browser and implement the resumable multithreading chunked data uploading. Additionally, the original genotyped SNP data can be stored in a remote cloud server, such as Google Drive. PIP-SNP provides the options to allow the user to provide the shared URL.

### Two venue interfaces for two specific application scenarios

LD bin mapping is the most crucial part of the process, but it is also very subtle, which will affect the whole implementation. Using the existing LD bin mapping information with higher accuracy usually is the top choice. Therefore, when we designed PIP-SNP, we considered fully the two practical application scenarios to directly detect the LD bins from the raw SNP data and to use the existing LD bin mapping information. Supplementary Figure S3 illustrates the diagram for the two scenarios. During the implementation in C/C++, we created two separate projects and compiled them into two executable command lines to handle the two distinct scenarios.

In the web client part, we populated the two scenarios separately as PIP_SNP_Venue1 and PIP_SNP_Venue2. Supplementary Figures S4–S7 are the snapshots of the web interface for the two application scenarios. PIP_SNP_Venue1 (Supplementary Figure S4) takes the raw SNP data as the only input (Supplementary Figure S3), while PIP_SNP_Venu2 (Supplementary Figure S6) requires two inputs, including the raw SNP data and the existing LD bin mapping information data (Supplementary Figure S3). After submitting, PIP_SNP_Venue1 will proceed with all the three processing procedures, and return two files, including the LD bin mapping result and the final SNP data preprocessing result (Supplementary Figure S5). PIP_SNP_Venue2 will skip the LD bin mapping step and proceed with the rest two processing procedures and return two files, including the updated LD bin mapping result and the final SNP data processing result (Supplementary Figure S7).

### User option to integrate additional processing as deep synthesizing

We investigated the correlation analysis of SNP data from the HapMap population and found that there may be more acute spike autocorrelation patterns. This phenomenon indicates that several types of SNPs may be closely entangled in a local region. We have developed a two-phase procedure, including a shallow- and deep-synthesizing step, to clump the SNPs into groups. Each group corresponds to a conceptual LD bin. However, the groups can overlap each other to some degree. If using deep synthesizing, we can achieve a higher SNP marker compression ratio, which will be defined and discussed in later sections. When we designed the architecture of PIP-SNP, we left to the user the option whether to choose one phase or two phases to generate the synthesized marker. When implementing this function module, we built and compiled a special project for processing the deep synthesizing. Supplementary Figure S8 illustrates the data flow chart among the three execute command lines.

To suit the two application scenarios, we needed to seam the three executable command lines into an integrative pipeline. In addition to developing the three executable command lines, we also developed some python scripts to seam the three executable command lines and parse the returned configured parameters from a remote client user.

## RESULTS

In this study, we have proposed a series of methods and developed a web-based pipeline PIP-SNP to preprocess the SNP data, including the LD conceptual bin mapping, missing value imputing and LD bin markers synthetizing. It was also important to address how much the genetic information has been conserved or lost due to the SNP markers being processed and greatly reduced. To address this question, we needed to go to the very nature of statistical genetic itself. Essentially, the genetic variants and the phenotypic values of quantitative traits can be connected through a kind of LMM. Statistically solving the proposed LMMs mainly include three procedures: kinship matrices calculation, genetic variance component analysis and statistical testing of *P-*values. A comprehensive comparison of the results of these three aspects can answer this question.

We developed a more complex LMMs and GWAS tool, PATOWAS, which can outperform the existing LMM and GWAS tools by delivering a specific broad-sense heritability, the marker's additive effect results and the marker pairs’ interaction effect results (13). Based on this specific LMM, we compared the results at the three aspects and evaluated the difference between using the full SNP data set and using the dimension-reduced synthetic markers. The data include a moderate-scale SNP dataset from rice RILs (36) and a high-dimensional SNP dataset from a rice HapMap project (30). The first dataset was used to demonstrate the proposed method and principles, the correlation and the auto-correlation characteristics of SNP signals, while the second dataset was used to demonstrate the challenge due to the high-dimension SNP markers and its solution. Table 1 shows the general information of two datasets, and Supplementary Table S1 shows further information about the high-dimensional SNP distribution across the 12 chromosomes.

**Table 1.** Summary information of the SNP dataset for performance evaluation

	SNP marker number	Individual number	Trait
Rice RIL Dataset	1619	210	YIELD
Rice HapMap Dataset	842,474	374	Days to Heading

### A LMM incorporating additive and interaction effects

The LMM that incorporates the markers’ additive effects and marker pairs’ interaction effects can be simply represented as

(6) $$\begin{eqnarray*} y &=& X\beta + \sum\nolimits_{i\ = {\rm{\ }}1}^M {{Z_i}{a_i}} \nonumber \\ && +\, \sum\nolimits_{i\ = {\rm{\ }}1}^{M - 1} {\sum\nolimits_{j\ = {\rm{\ }}i + 1}^M {\left( {{Z_i}\# {Z_j}} \right){{\left( {aa} \right)}_{ij}} + e} } \end{eqnarray*}$$

where *y* is an *N* × 1 vector of a quantitative phenotypic trait, and *Z* is an *M* × *N* marker matrix for *M* SNP markers and *N* individual samples. $X\beta$ is the intercept; ${Z_i}$ is the *i*th column of matrix *Z*, and *a_i_* is the *i*th marker’s additive effect on the trait; ${Z_i}\# {Z_j}$ is element-wise product of vectors ${Z_i}$ and ${Z_j}$, and ${( {aa} )_{ij}}$ is the interaction effect between marker *i* and marker *j*; *e* is an *N* × 1 vector of residual error.

### The variance of phenotypic trait *y* can be represented as

(7) $$\begin{equation*}Var \left( y \right) = {K_a} \sigma _a^2 + {K_{aa}}\sigma _{aa}^2 + I{\sigma ^2}\end{equation*}$$

where ${K_a}$ and ${K_{aa}}$ are additive and interaction effect kinship matrix respectively; and $\sigma _a^2$, $\sigma _{aa}^2$ and ${\sigma ^2}$ are the variance components to be estimated for additive effect, interaction effect and residual, respectively. More details for the two kinship matrix calculations and the three variance estimations can be referred to our published pipeline PATOWAS (13). Based on the three estimated variance components, the broad-sense heritability representing how much the biologically explainable genetic components can be calculated by

(8) $$\begin{equation*}H = \frac{{\sigma _a^2 + \sigma _{aa}^2}}{{\sigma _a^2 + \sigma _{aa}^2 + {\sigma ^2}}} \end{equation*}$$

PATOWAS also output the testing results as p values measuring how likely the putative trait associated with genetic variants as SNP markers or SNP marker pairs is due to random chance.

Using PIP-SNP, we can configure different parameters to get a dimension reduced synthetic marker. Compared with the original high-dimensional SNP markers, the marker compression ratio (MCR) as an analogy of compression ratio in image processing can be defined as

(9) $$\begin{equation*}{\rm MCR }= \frac{{{\rm No.}\ {\rm of}\ {\rm the}\ {\rm original}\ {\rm high}\ {\rm dimensional}\ {\rm SNP}}}{{{\rm No}.\ {\rm of}\ {\rm the}\ {\rm synthesized}\ {\rm marker}{s}}} \end{equation*}$$

We submitted the two typical SNP datasets, including a RIL population and a HapMap population, to PIP-SNP and generated a series of dimension-reduced synthesized SNPs or markers. Table 2 show a summary of the dimension-reduced markers by PIP-SNP.

**Table 2.** Summary information of the dimension-reduced markers from PIP-SNP

Cutoff $R\_th$		0.8	0.6	0.4	0.2
Rice RIL	No. of shallow synthesized markers	196	103	65	43
	MCR	8.2602	15.7184	24.9077	37.6512
Rice HapMap	No. of deep synthesized markers	339,493	254,289	186,174	115,544
	MCR	2.4816	3.3131	4.5252	7.2914

**Figure 5.**
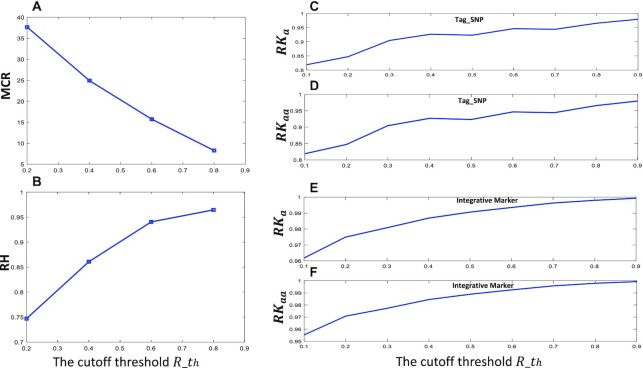
The perspective of the marker reduction and its effect on the heritability and kinship matrix. At different cutoff thresholds$\ R\_th$, the evaluation plots are generated. (**A**) Marker compression ratio (MCR), (**B**) relative heritability (RH), (**C**–**F**) correlation of the kinship matrix *K*a, *K*aa between that were generated by full markers and that were generated by reduced markers either as the tag SNPs (C and D) or as the integrative markers (E and F).

**Figure 6.**
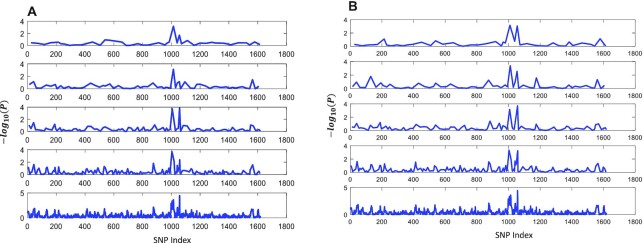
The aligned line sub-plots of the negative log10 ($P{\rm{\ }}$values) resulted at two typical synthesizing modes. (**A**) The aligned subplots for the *P* values generated in the synthesizing mode of ‘Representative tag SNP’. (**B**) The aligned subplots for the *P* values generated at the synthesizing mode of ‘Integrating all SNPs in the Bin’. From top to bottom, the cutoff thresholds $R\_th$ were set at 0.2, 0.4, 0.6, 0.8 and 1.0, respectively.

We then submitted the marker data, together with the phenotype trait data, to PATOWAS for the genetic performance evaluation. Kinship matrix measures the relatedness between individuals, and its accuracy will affect the following p value testing. Epistatic GWAS needs to calculate two kinds of kinship matrices ${K_a}$ and ${K_{aa}}$, which have the complexity of $O(M{N^2}$), $O({M^2}{N^2}$), respectively (12,13). Therefore, the calculation of kinship matrix will cost a huge computation burden. In the following sections, we first used the moderate-scale SNP data from a RIL rice population and its dimension-reduced SNPs/markers (Table 2) to demonstrate the evaluation results of kinship matrix, broad-sense heritability and the 1D Manhattan plot. All the results are shown in Figures 5 and 6. We also analyzed the high-dimensional SNP data from a rice HapMap population but found a challenge to achieve a higher MCR even at a very low correlation cutoff threshold $R\_th$. However, if we choose the deep-synthesizing method and set the cutoff threshold $R\_th$ at 0.2, we could achieve ∼7.0 times marker compression ratio (Table 2).

### Evaluation of the resulted kinship matrix

A kinship matrix essentially measures the relatedness of individuals. Considering its symmetric feature, a kinship matrix can be represented as a lower triangular matrix. Therefore, the additive and interaction effect kinship matrix ${K_a}$ and ${K_{aa}}$can be represented as formulas 10 and 11, respectively.

(10) $$\begin{equation*}{K_a} = \left[ {\begin{array}{@{}*{3}{c}@{}} {{K_a}\left( {1,1} \right)}&\ &\ \\ {{K_a}\left( {2,1} \right)}&{{K_a}\left( {2,2} \right)}&\ \\ {\begin{array}{@{}*{1}{c}@{}} \vdots \\ {{K_a}\left( {N,1} \right)} \end{array}}&{\begin{array}{@{}*{1}{c}@{}} \ddots \\ \cdots \end{array}}&{\begin{array}{@{}*{1}{c}@{}} \ddots \\ {{K_a}\left( {N,N} \right)} \end{array}} \end{array}} \right]\ \end{equation*}$$

(11) $$\begin{equation*}{K_{aa}} = \left[ {\begin{array}{@{}*{3}{c}@{}} {{K_{aa}}\left( {1,1} \right)}&\ &\ \\ {{K_{aa}}\left( {2,1} \right)}&{{K_{aa}}\left( {2,2} \right)}&\ \\ {\begin{array}{@{}*{1}{c}@{}} \vdots \\ {{K_{aa}}\left( {N,1} \right)} \end{array}}&{\begin{array}{@{}*{1}{c}@{}} \ddots \\ \cdots \end{array}}&{\begin{array}{@{}*{1}{c}@{}} \ddots \\ {{K_{aa}}\left( {N,N} \right)} \end{array}} \end{array}} \right]\ \end{equation*}$$

The two kinship matrices ${K_a}$ and ${K_{aa}}$ can be dumped into two one-dimensional kinship vectors $\widehat {{K_a}}$, $\widehat {{K_{aa}}}$ as formulae 12 and 13.

(12) $$\begin{eqnarray*} \widehat {{K_a}} &=& \left[ {K_a}\left( {1,1} \right),{K_a}\left( {2,1} \right),{K_a}\left( {2,2} \right), \ldots, \right. \nonumber \\ && \left. {K_a}\left( {N,1} \right), \ldots ,{K_a}\left( {N,N} \right) \right] \end{eqnarray*}$$

(13) $$\begin{eqnarray*}\widehat {{K_{aa}}} &=& \left[ {K_{aa}}\left( {1,1} \right),{K_{aa}}\left( {2,1} \right),{K_{aa}}\left( {2,2} \right), \ldots , \right. \nonumber \\ && \left. {K_{aa}}\left( {N,1} \right), \ldots ,{K_{aa}}\left( {N,N} \right) \right] \end{eqnarray*}$$

If we set a correlation cutoff threshold $R\_th$, PIP-SNP will map all the SNPs into blocks and partition them into LD conceptual bins, then output dimension-reduced synthetic markers. The two corresponding 1D kinship vectors are represented as $\widehat {{K_a}}( {R\_th} )$ and $\widehat {{K_{aa}}}( {R\_th} )$, respectively. Then two correlation coefficients measuring the kinship matrix similarity between $\widehat {{K_a}}( {R\_th} )$ and $\widehat {{K_{aa}}}( {{R_{th}}} )$ using the synthetic markers against $\widehat {{K_a}}$, $\widehat {{K_{aa}}}$ and using the original high-dimensional SNP markers can be calculated by the formulae 14 and 15.

(14) $$\begin{equation*}R{K_a}\left( {R\_th} \right) = Corr\left( {\widehat {{K_a}},\ \widehat {{K_a}}\left( {R\_th} \right)} \right)\end{equation*}$$

(15) $$\begin{equation*}R{K_{aa}}\left( {R\_th} \right) = Corr\left( {\widehat {{K_{aa}}},\ \widehat {{K_{aa}}}\left( {R\_th} \right)} \right)\end{equation*}$$

The representative marker for each bin can be the optimal tag SNP or the integration of all SNPs. Based on the PATOWAS analysis result for the RIL population SNP data, Figure 5C–F illustrate the correlation measurements of the two kinship matrices using the original SNP markers and the synthesized markers at cutoff threshold $R\_th$. From Figure 5, we can see that the kinship matrix similarity moderately decreases with the decreasing cutoff threshold $R\_th$ and the synthesized marker number is reduced. On the other side, we set the cutoff threshold $R\_th$ as low as 0.2 and the marker compression ratio MCR can reach 37.7, but the kinship matrix similarity using integrative marker is still as high as 0.96 (Figure 5A, E and F). These phenomena indicate that there is, indeed, information redundancy among SNPs.

Comparably, the integration method considers all the SNP information in one detected LD bin. Therefore, this method conserves the more genetic information and reports the higher kinship matrix similarity. The mathematical bases that prove this characteristic have been detailed and deduced in Supplementary Note S4. For the HapMap population data, if we choose the deep-synthesizing method, a high kinship matrix similarity of 0.9 could be achieved in the case of ∼7.0 times SNP marker compression ratio (Supplementary Table S2).

### Evaluation of the resulted broad sense heritability

Based on the PATOWAS result, we can use formula 8 to calculate the broad-sense heritability. Both the narrow and broad sense heritability can be used to measure the portion of the phenotypic variation that can be biologically explained by the considered causal genetic variants. Comparably, the broad-sense heritability delivers more explainable genetic components because the canonical narrow-sense heritability considers only the marker's additive effect.

The two calculated broad-sense heritability using the original high dimensional SNP data and dimension reduced synthetic marker are represented as ${H_0}$ and $H( {R\_th} )$ respectively, then we can use formula 16 to define a relative heritability (RH) to measure how much the genetic information has been conserved during the SNP data dimension-reduced processing.

(16) $$\begin{equation*}RH \left( {R\_th} \right) = \frac{{H\left( {R\_th } \right)}}{{{H_0}}}\ \end{equation*}$$

To a specific trait, the defined RH is can be greatly affected by the dimension reduced SNP markers. Based on the analysis result for RIL data, we generated Figure 5B to demonstrate the relationship between the $RH$ and the correlation cutoff threshold $R\_th$. With the $R\_th$ decreasing, the MCR increase and the $RH$ decrease slowly. Even when we set the $R\_th$ as low as 0.2 and MCR at 37.7, the $RH$ can be 0.75. This means that only 1/37.7 = 2.7% markers can retain 75% of the original genetic information. Again, this phenomenon indicates the LD block structure of the genome and the information redundancy among SNPs.

### Comparison of the line Manhattan plots of $ - lo{g_{10}}( {P\ values} )$

GWAS analysis needs the genotype and phenotype data as inputs, and the GWAS analysis usually delivers the $P\ values$ as the probability assuming the null hypothesis is correct. Therefore, the corresponding $ - lo{g_{10}}( {P\ value} )$ can be used to measure how much the SNP marker is relevant to the trait to be investigated. For very dense SNP markers, we usually use a type of scatter plot called Manhattan plot to display a large number of *P*-value points. In this study, we developed pre-processing methods and platforms to group and synthesize SNP markers, which will cause the marker number to be reduced and make it easier to be manipulated in the GWAS analysis. However, biologists and breeders may show more concern regarding whether the significant QTL patterns can be conserved.

Based on the PATOWAS analysis result for the moderate-scale RIL data, we generated a series of aligned line plots of $ - lo{g_{10}}( {P\ values} )$. Here, the marker numbers are continuously synthesized from 1619 to 43 with a different correlation cutoff threshold $R\_th$ at two synthesizing modes (Figure 6). From Figure 6, we can see that most of the QTL patterns have been well conserved. For the very high-dimensional SNP data from a HapMap population, we first performed GWAS analysis using classical LMM in TASSEL and our in-house PATOWAS with the configuration to bypass scanning the p values for interaction effect. The resulted two separate 1D GWAS results are illustrated in Supplementary Figures S9 and S10. We can see that the QTL patterns in the two Manhattan plots are very similar.

We then submitted the dimension-reduced tag SNP markers to both TASSEL and PATWOAS. TASSEL has the option to accept user-defined kinship matrix. Therefore, we have two types of TASSEL results corresponding to the kinship matrix from the full SNP marker set or from dimension reduced markers. The 1D GWAS result, including the aligned Manhattan plots and the *Q*–*Q* plots at different correlation cutoff threshold $R\_th$, are presented in Supplementary Figures S11–S22. From Supplementary Figures S11–S22, we can see that the decreased synthesized marker number only slightly deflated the $P\ values$ but well conserved nearly all of the major QTL patterns. Comparing Supplementary Figure S17 vs Supplementary Figure S18, and Supplementary Figure S20 vs Supplementary Figure S21, we can conclude that adopting the full marker resulted kinship matrix do not improve but may degrade the association resolution.

This phenomenon can be explained by the theoretical basis of the Beavis effect describing the relationship between independent QTL number and the sample size (37).

## DISCUSSION

Due to the existence of LD and haplotype block patterns, the SNPs are not independent and the whole genome can be mapped into block structures. SNP data face two obvious challenges: the huge computing burden due to its high dimensionality and the more missing values affecting the biological completeness. SNP data need to be processed before conducting the downstream GWAS analysis. In this study, we used stochastic processes to describe the SNP signals and proposed two kinds of autocorrelation to measure the information redundancy of nearby SNPs. Based on the autocorrelation measures, we proposed novel methods to detect the LD conceptual bins. Further, we treated each detected bin independently and used the kNN method to infer the missing values. Finally, one representative marker per LD conceptual bin can be synthesized either as the optimal tag SNP or the integrative marker using the Euclidean norm of all SNPs.

The dimension-reduced synthetic markers will inevitably cause genetic information loss, yet the marker dimension reduction is necessary for epistatic GWAS analysis. To address how much the genetic information is conserved or lost due to the preprocessing of SNP markers, we used our in-house association tool, namely PATOWAS, to evaluate the resulted relative heritability, kinship matrix and the canonical 1D Manhattan plots. The defined relative broad sense heritability includes two biological components for additive effect and interaction effect. Therefore, it is possible that the relative broad sense heritability does not reduce so much, but the 1D Manhattan plot becomes noisy, and the QTL patterns become less evident.

We analyzed two kinds of typical SNP data, including a moderate-scale SNP dataset from a RIL rice population and a high-dimensional SNP dataset from a rice HapMap population. We found that it is more challenging to describe the random SNP data from a HapMap population. The autocorrelation criteria spreading across the neighboring two SNPs is very limited to grouping similar SNPs and, therefore, it cannot achieve a satisfactory SNP marker compression ratio. However, the very acute spike autocorrelation patterns indicate that several kind of SNPs can be closely entangled together in a local region, which inspire us to jump out off the neighbor-joining SNPs and consider its second and even third neighbor SNPs. Based on these thinking, we developed a specific function module called deep synthesizing, which is more aggressive in grouping the more neighbor SNPs and distinguishes from the shallow synthesizing method by spreading consideration of only the two neighbor-joining SNPs.

To the detected LD bins, we propose two options to generate the representative synthetic marker. One is to find an optimal tag SNP, and the other is to calculate the Euclidean norm of all SNPs to get an integrative marker. The first option can keep the same genetic variant format and the marker resolution but will lose the genetic information from other SNPs. Comparably, the second option considers the integrative genetic information of all the SNPs at the same LD bin. Our performance evaluation results also support that more genetic information has been retained. However, the genetic variant data format will be changed into float, and the marker resolution will be degraded from a single SNP into a LD conceptual bin.

We realized that LD bin mapping is the most important part compared with other modules. The actual bin mapping should match well to the real haplotype block structure and reflect the genetic recombination. However, the actual genetic recombination event is difficult to know and different haplotype block partitioning algorithms produce a varied range in the bin number, size and coverage, which make it difficult to propose a golden standard to define the haplotype block and then conduct a fair performance evaluation (22). As such, the LD bin mapping is very subtle, which may affect other processing modules and the downstream GWAS analysis. To be flexible, we considered two possible application scenarios and designed two venue interfaces as PIP_SNP_Venue1 and PIP_SNP_Venue2. Users have the option to use their own confident LD bin mapping results for the missing genotype imputing and/or the synthesis marker generating. Further, if the user provides a fixed size (e.g. integer $l$) LD bin mapping file and also specific an integer $k$ as the kNN method, the processing module for missing value imputation will be equal to the LD-kNNi method with the same parameters used in the tool LinkImpute (Money *et al.*, 2015).

Although methods have been developed to perform haplotype block mapping, imputing or tag SNP selection. However, most of them were independently developed for a specific aim. It's difficult to integrate them into a pipeline for high dimensional SNP data manipulation (Supplementary Table S3). To the best of our knowledge, there is no tool/platform that can implement all the three function modules in a one-stop processing.

## DATA AVAILABILITY

The pipeline PIP-SNP, source codes for three project packages PIP_SNP_Venue1, PIP_SNP_Venue2 and Deep Synthesizing, test data, including a RIL rice data, HapMap rice data and phenotypic traits, are freely available at https://bioinfo.noble.org/PIP_SNP/. We are committed to maintaining and improving the specific function modules per user comments and suggestions. Additionally, we have made the source code open and deposited them in Git-Hub https://github.com/noble-research-institute/PIP_SNP.

The current version of PIP-SNP only accepts biallelic SNP data in pure text format. The SNP data must be stored as $M \times N$ matrix and the genotype value numerically coded as 0 for homozygous major allele, 1 for heterozygous allele, 2 for homozygous minor allele, and -1 for the missing value to be imputed. In the future, we plan to develop additional modules for numerical genotype coding that can directly support the sequence SNP data as format of vcf or HapMap. We believe that such developments will provide much convenience to the users.

## Supplementary Material

lqab060_Supplemental_Files
