# Supplementary material for: PIP-SNP: a pipeline for processing SNP data featured as linkage disequilibrium bin mapping, genotype imputing and marker synthesizing
Source: NAR Genom Bioinform. 2021 Jul 5;3(3):lqab060. doi: 10.1093/nargab/lqab060 (PMC8256826; doi:10.1093/nargab/lqab060)
Supplement: lqab060_Supplemental_Files [file lqab060_supplemental_files.zip › PIP_SNP_Supplemental_Figures.pdf]

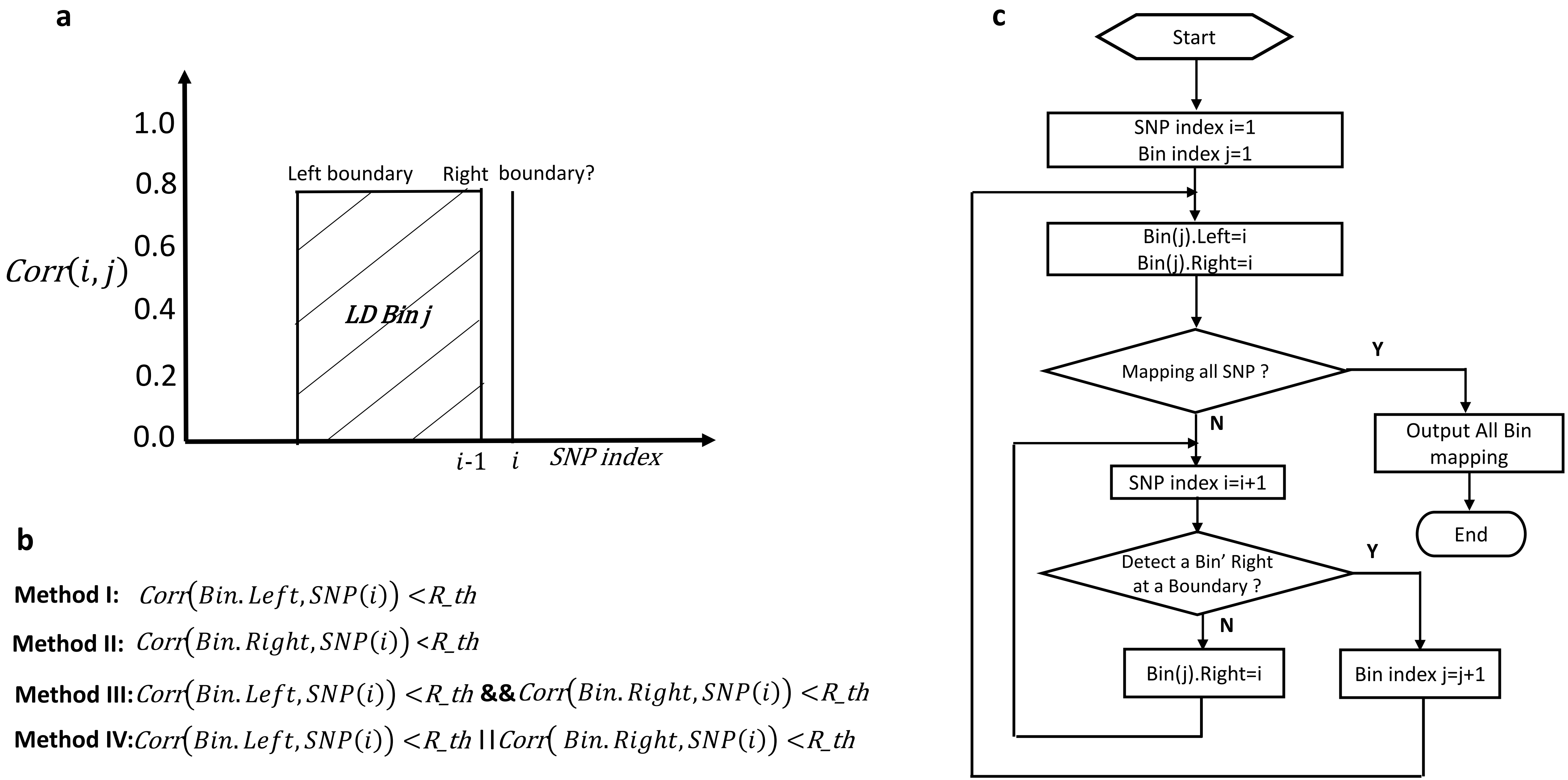

Supplementary Figure S1. The illustration of LD Bin detecting and mapping in PIP-SNP. (a.) LD Bin is defined by it's initial left boundary and right boundary detected at a breakthrough point. (b.) Four methods to detect a LD bin's right boundary, which is based on the comparison of the correlation between the SNP(i) at an assumed breakthrough point with a Bin's Left and/or Right. (c.) The flowchart of LD bin's detecting and mapping across all of SNPs.

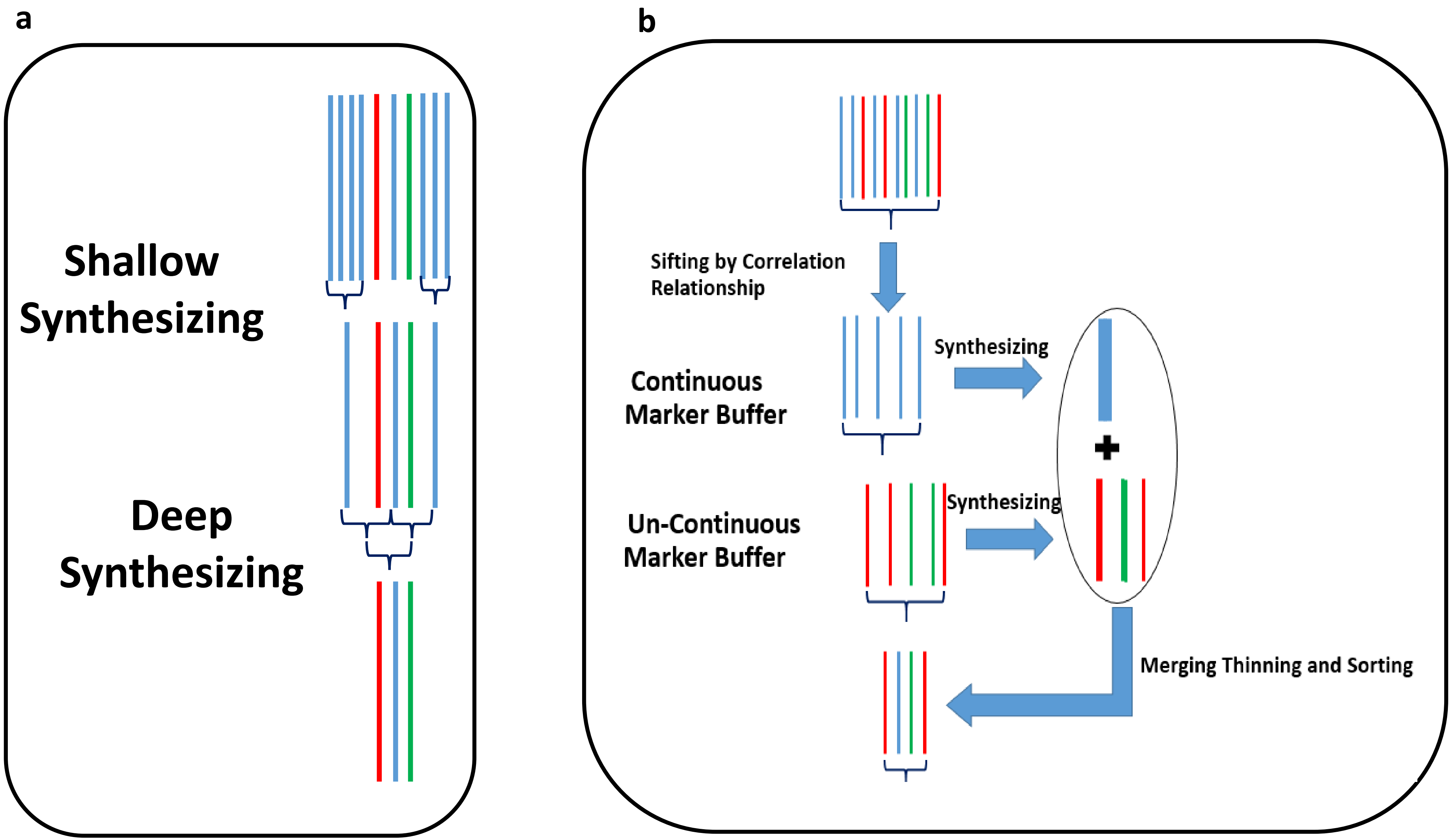

Supplementary Figure S2. Illustration to the deep synthesizing when preprocessing the more changing SNP data.
(a) Cascade relationship between shallow synthesizing and deep synthesizing. (b) Implementation flowchart of deep synthesizing

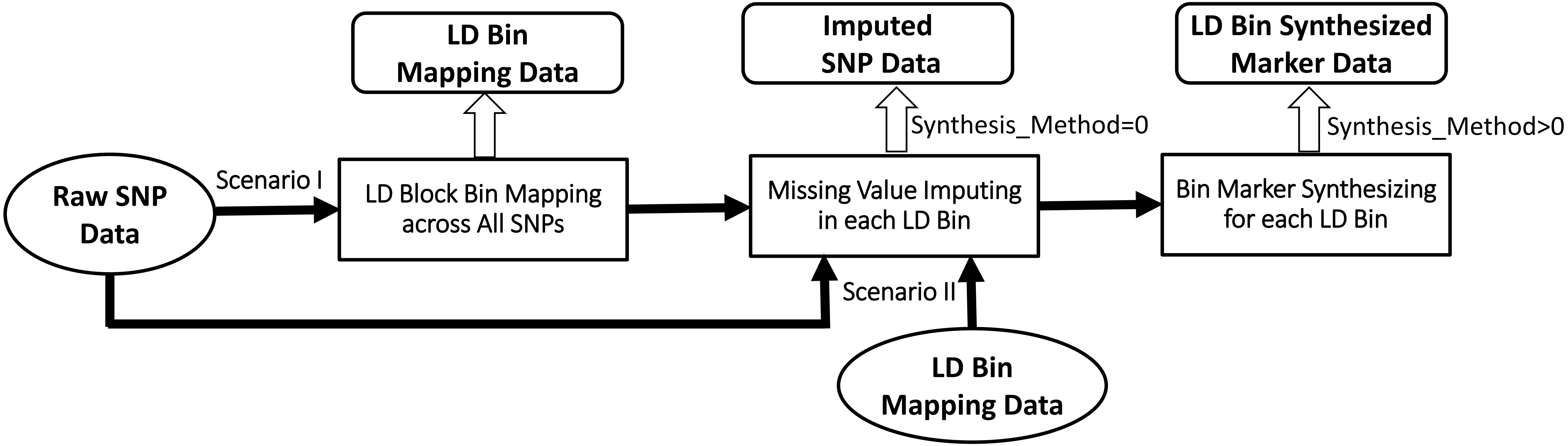

Supplementary Figure S3. The flowchart of PIP-SNP and its two typical application scenarios as without / with pre-defined LD Bin mapping data. **Scenario I** take raw SNP data as the only input and includes 3 processing modules as LD bin mapping, missing value imputing, and Bin marker synthesizing. **Scenario II** need the raw SNP data and the pre-defined LD Bin mapping data as input, but only contain two processing modules.

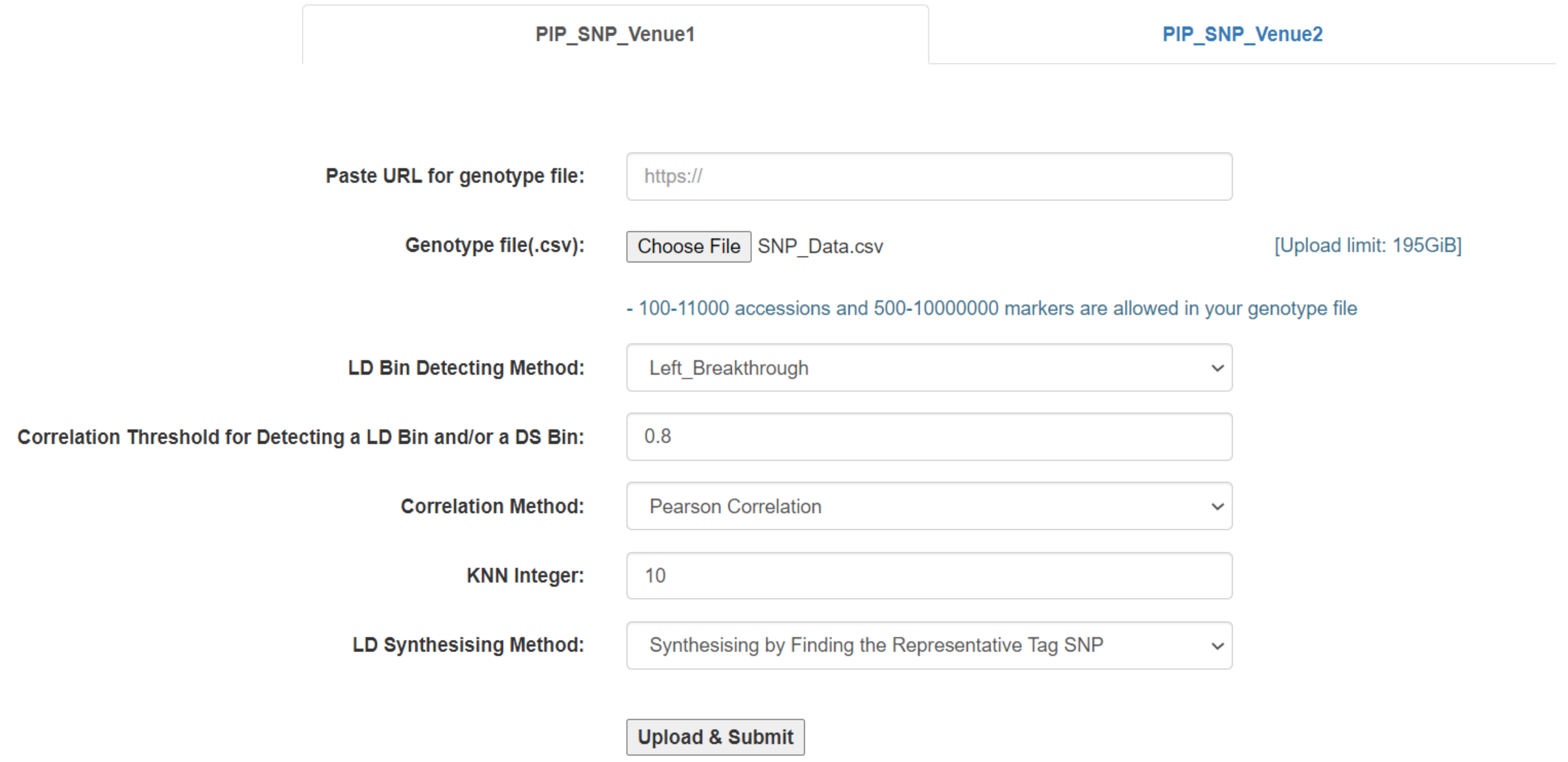

Supplementary Figure S4. User interface of PIP_SNP at the venue 1 requiring only the genotypic SNP data as input.

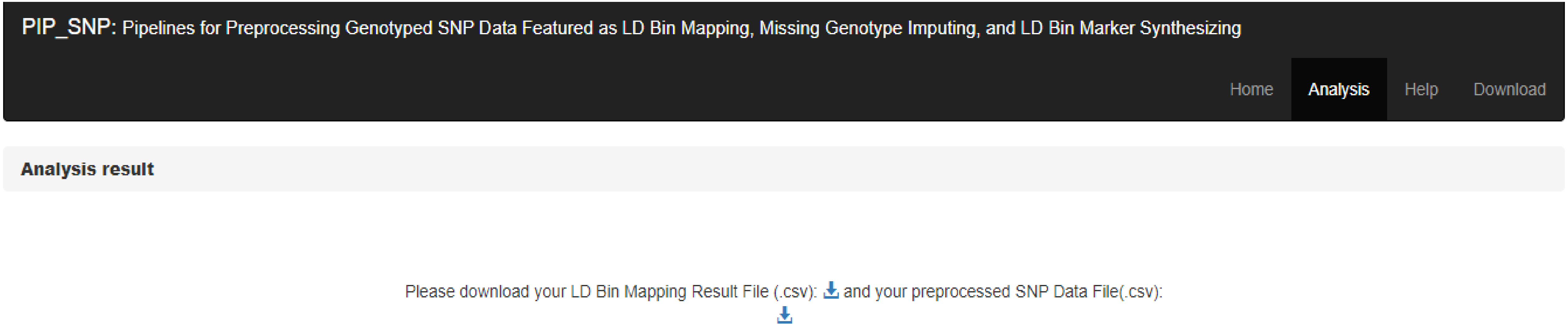

Supplementary Figure S5. Interface of PIP-SNP at the venue 1 for user to download the analysis result.

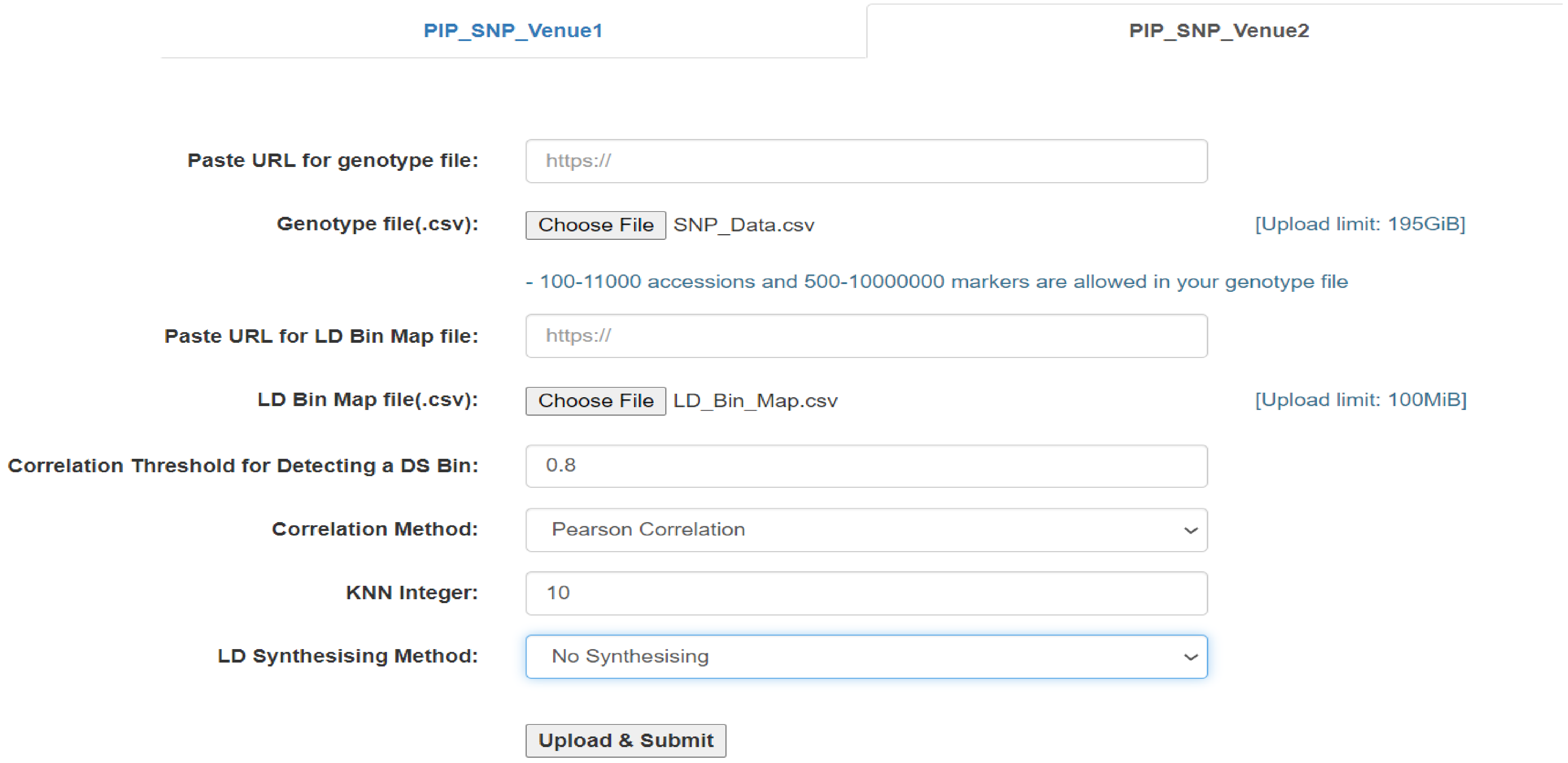

Supplementary Figure S6. User interface of PIP-SNP at the venue 2 requiring both the genotypic SNP data and LD bin map data as input.

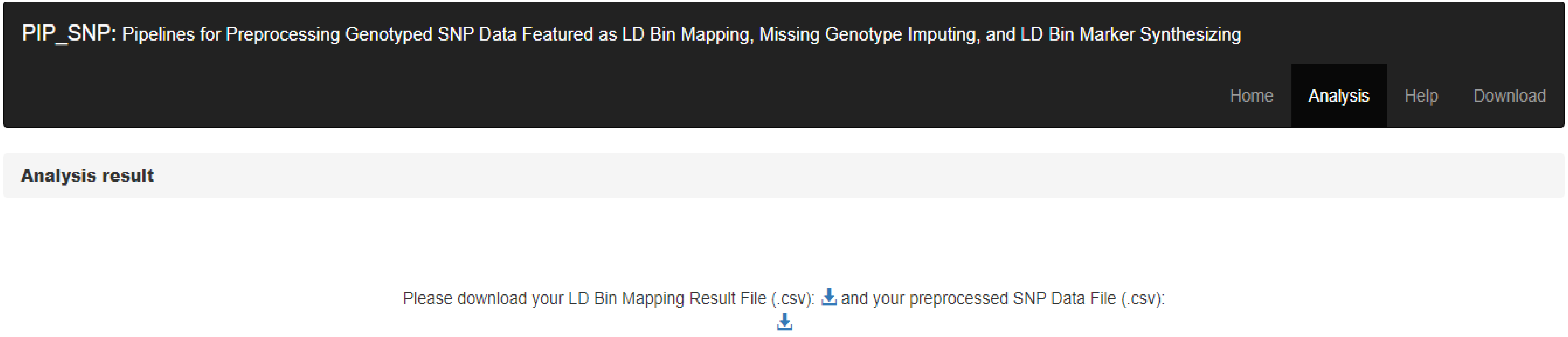

Supplementary Figure S7. Interface of PIP-SNP at the venue 2 for user to download the analysis result.

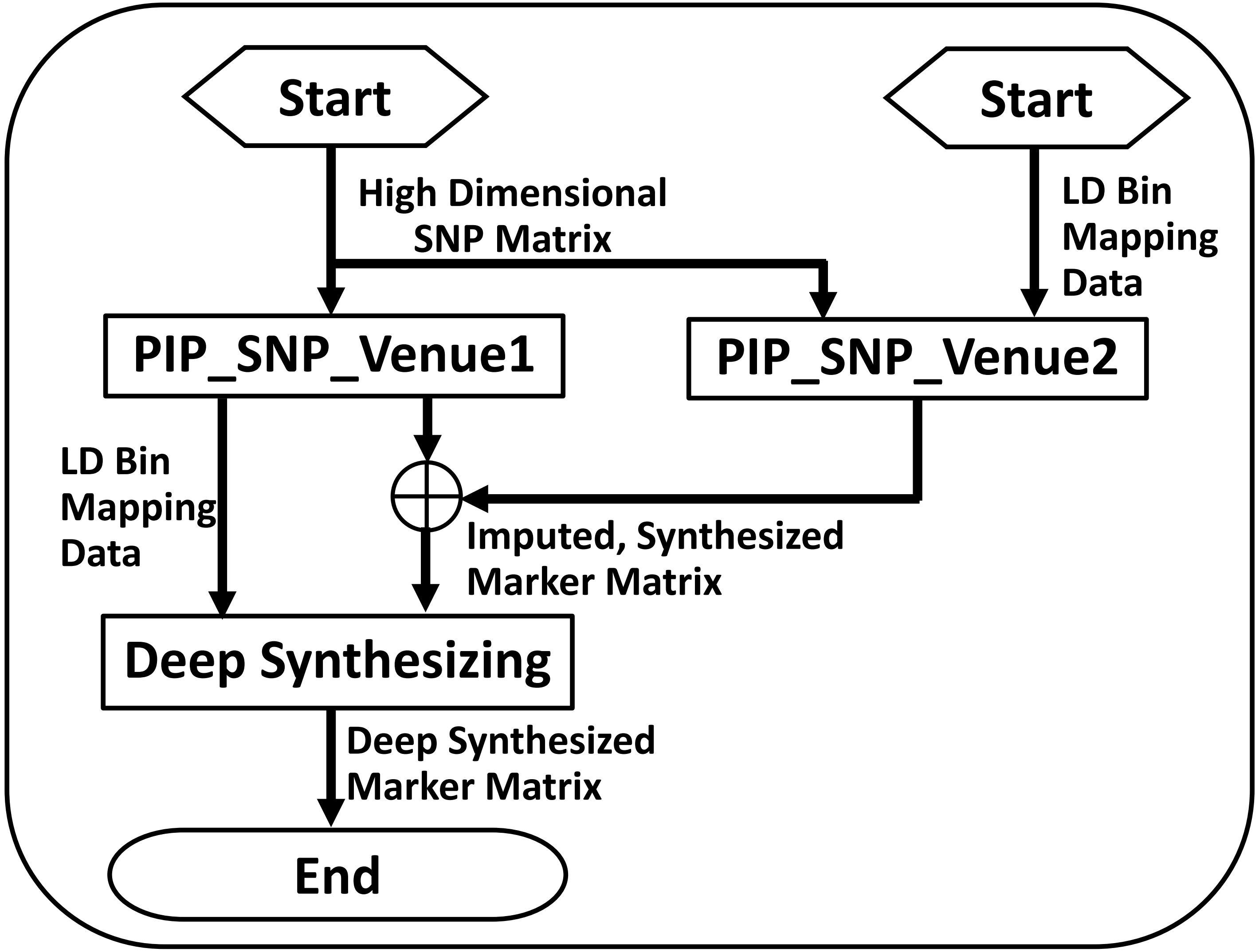

Supplementary Figure S8. Flowchart to process SNP data at two typical application scenarios and three executable projects.

a

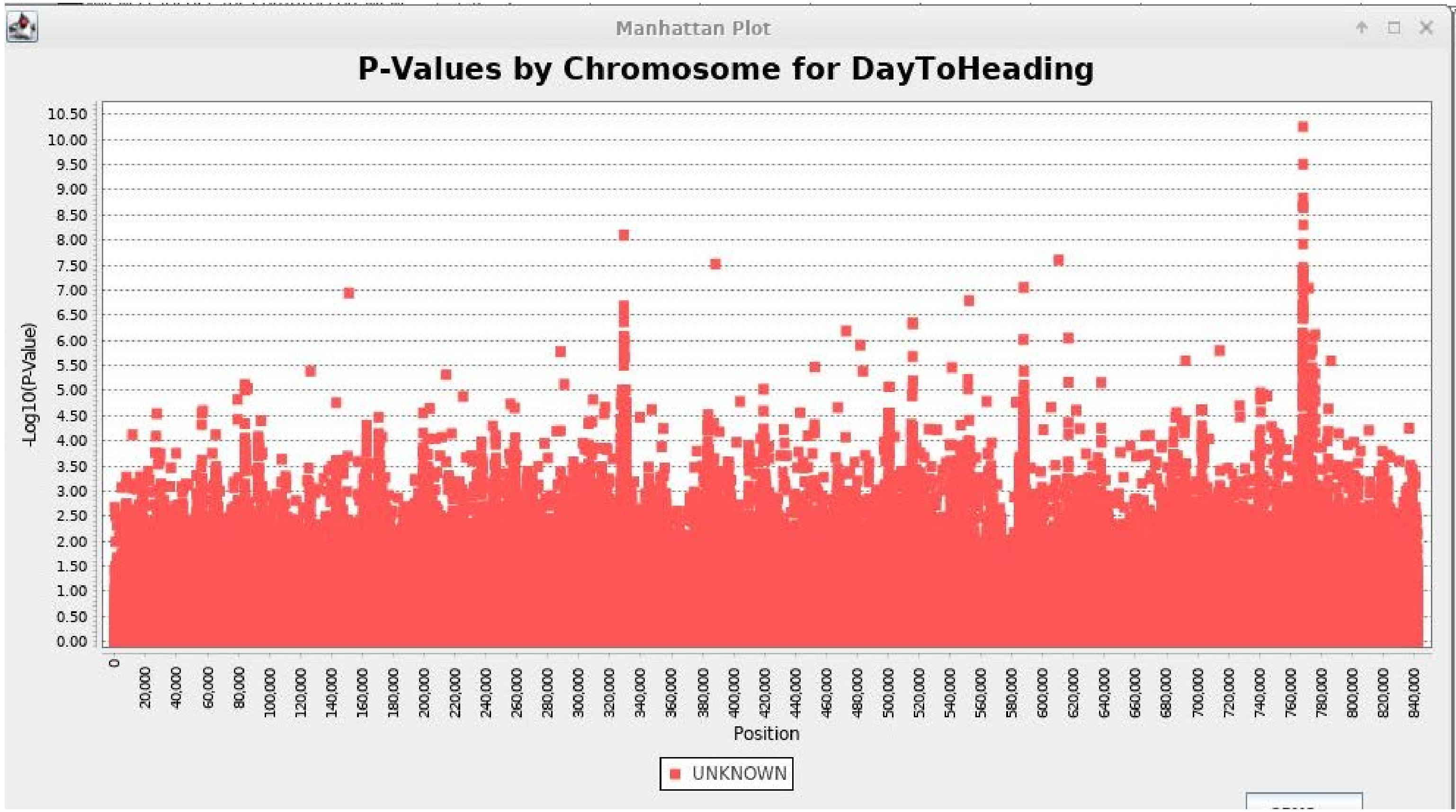

b

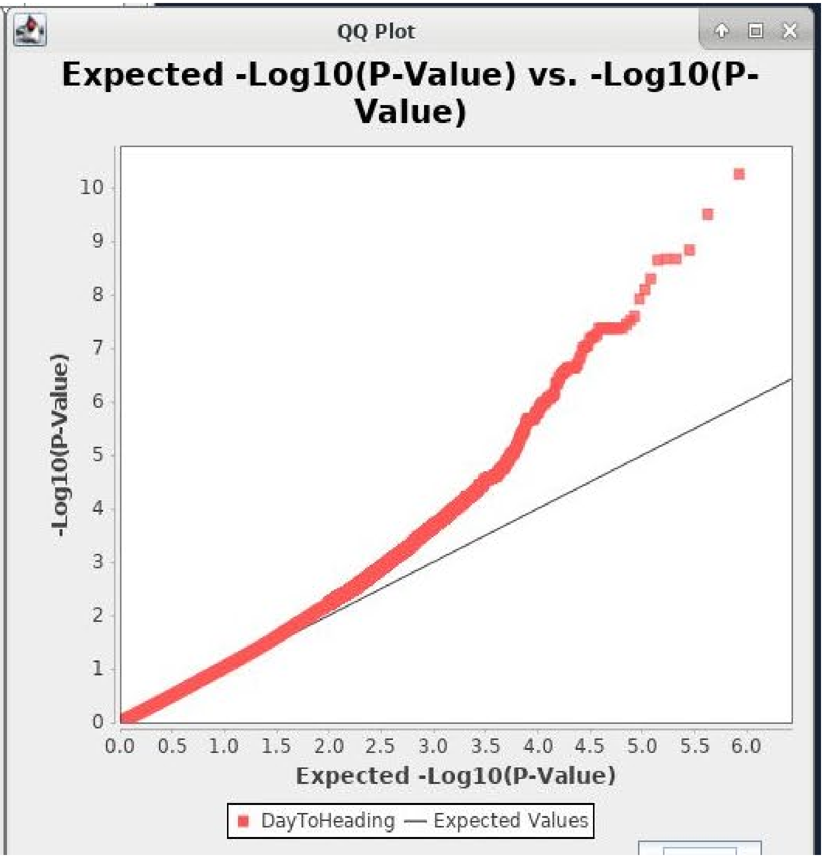

Supplementary Figure S9. The illustration of GWAS result based on the TASSEL analysis for the full 842,474 SNPs. (a.) Manhattan plot. (b.) Q-Q plot

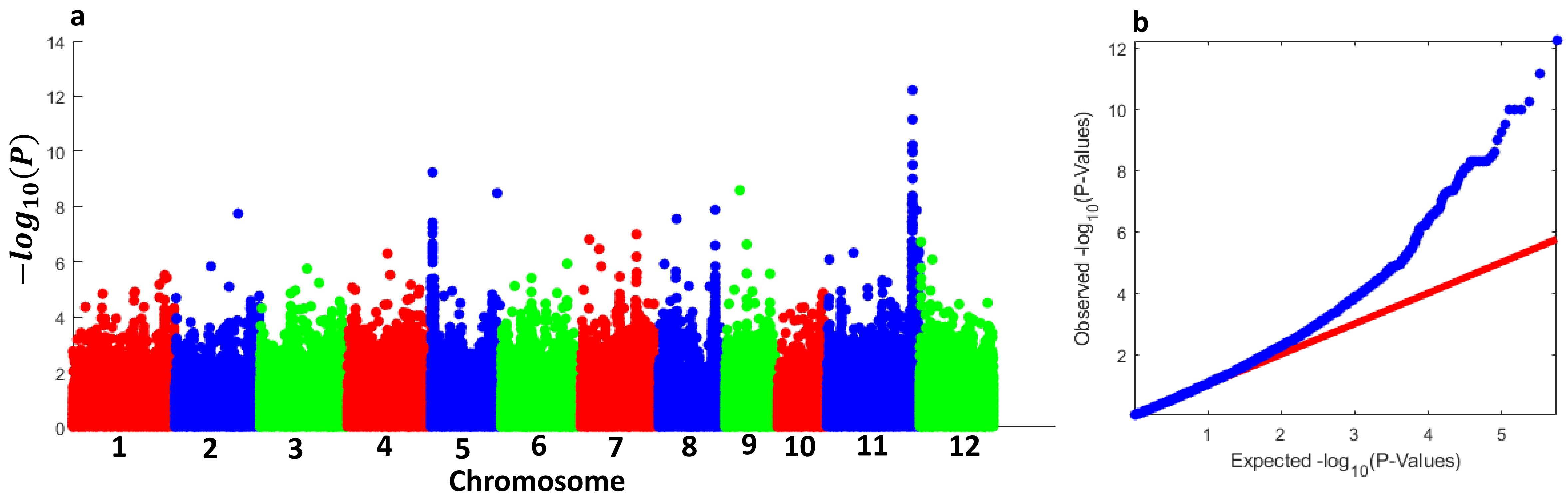

Supplementary Figure S10. The illustration of 1D GWAS result based on the PATOWAS analysis for the full 842,474 SNPs. (a.) Manhattan plot. (b.)Q-Q plot.

a b

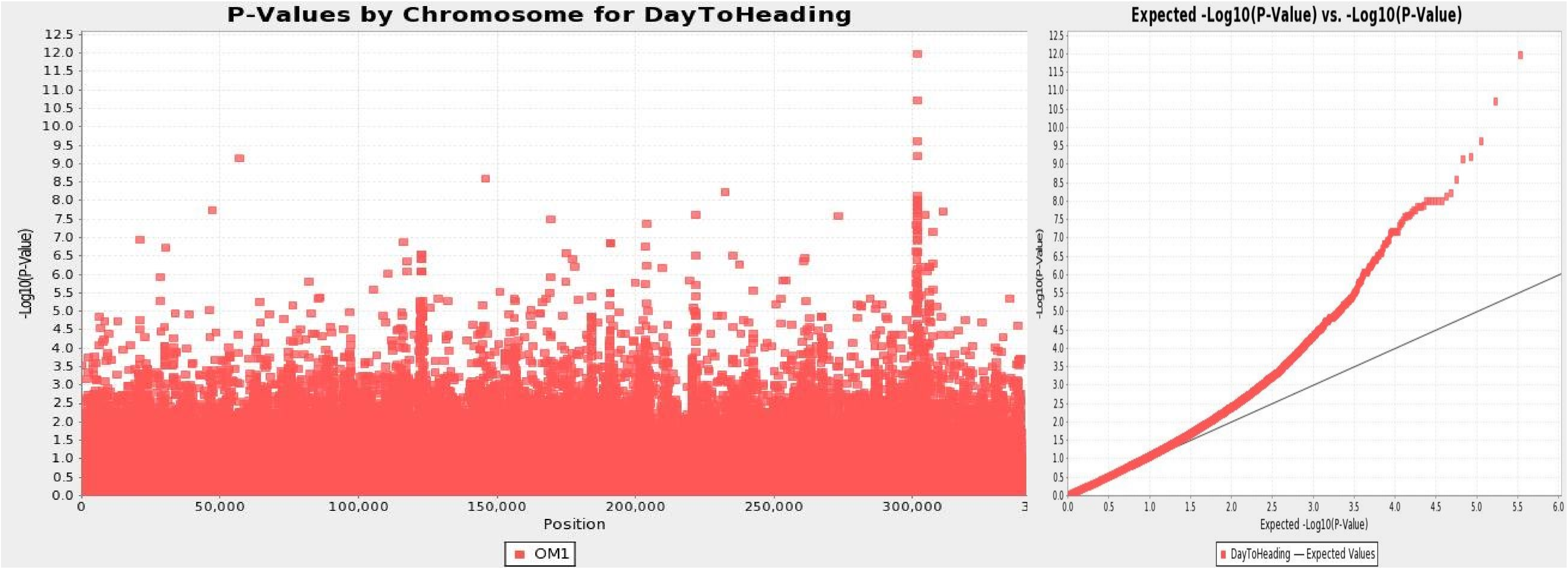

Supplementary Figure S11. The illustration of GWAS result based on the TASSEL analysis using the full 842,474 SNPs resulted kinship matrix and 339,493 synthesized SNPs. The synthesized SNPs are generated from PIP-SNP when configuring the cutoff threshold R_th as 0.8 and choosing "Deep synthesizing by finding the representative Tag SNP. (a.) Manhattan plot. (b.) Q-Q plot

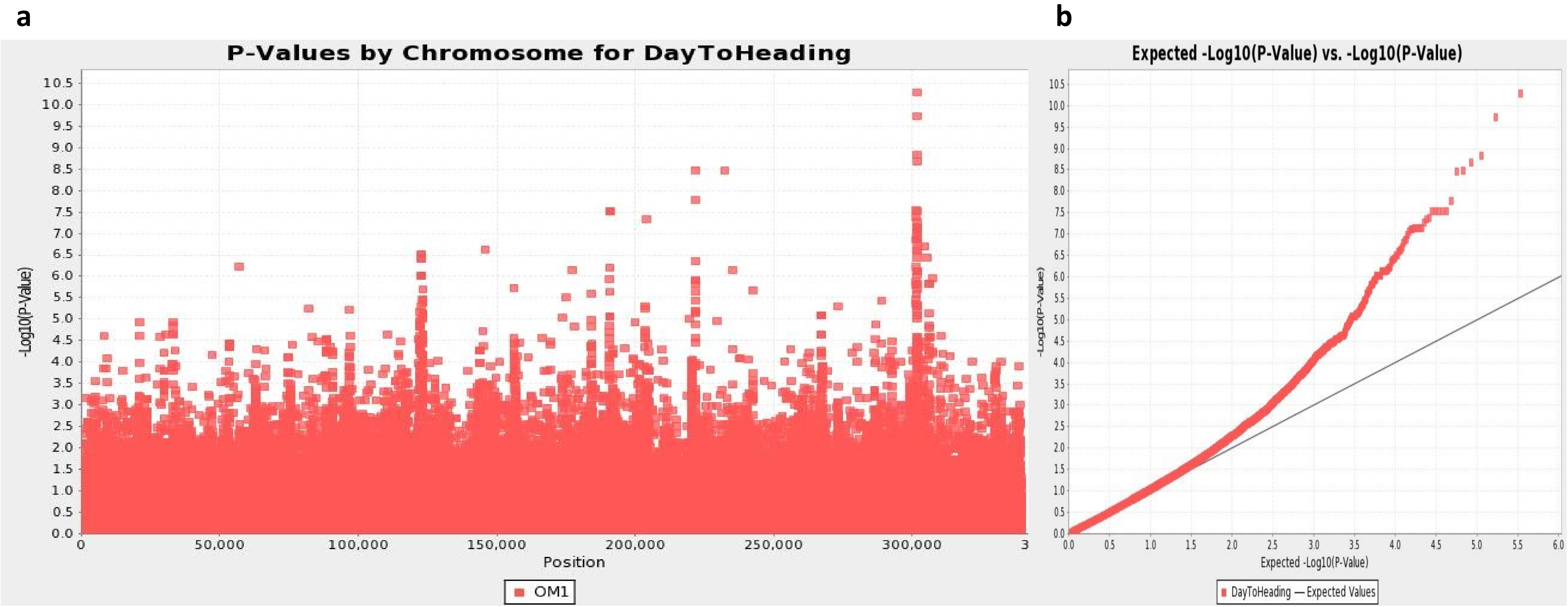

Supplementary Figure S12. The illustration of GWAS result based on the TASSEL analysis using 339,493 synthesized SNPs. The synthesized SNPs are generated from PIP-SNP when configuring the cutoff threshold R_th as 0.8 and choosing "Deep synthesizing by finding the representative Tag SNP. (a.) Manhattan plot. (b.) Q-Q plot

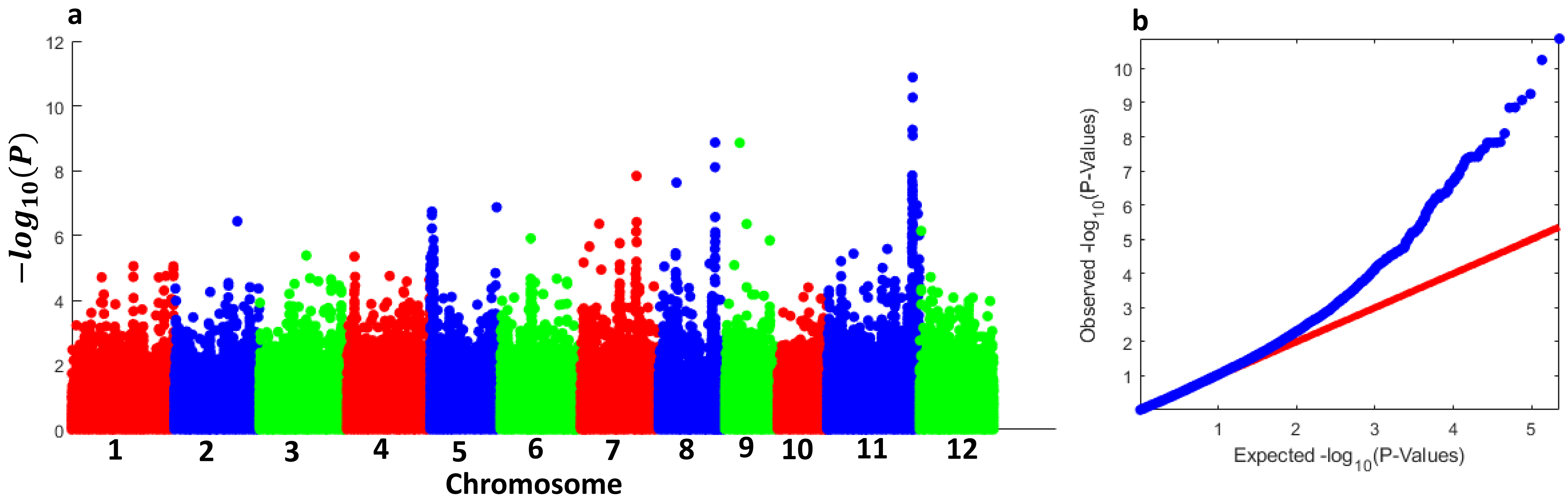

Supplementary Figure S13. The illustration of 1D GWAS result based on the PATOWAS analysis for the 339,493 synthesized SNPs from PIP-SNP when configuring the cutoff threshold R_th as 0.8 and choosing “Deep synthesizing by finding the representative Tag SNP”. (a.) Manhattan plot. (b.) Q-Q plot.

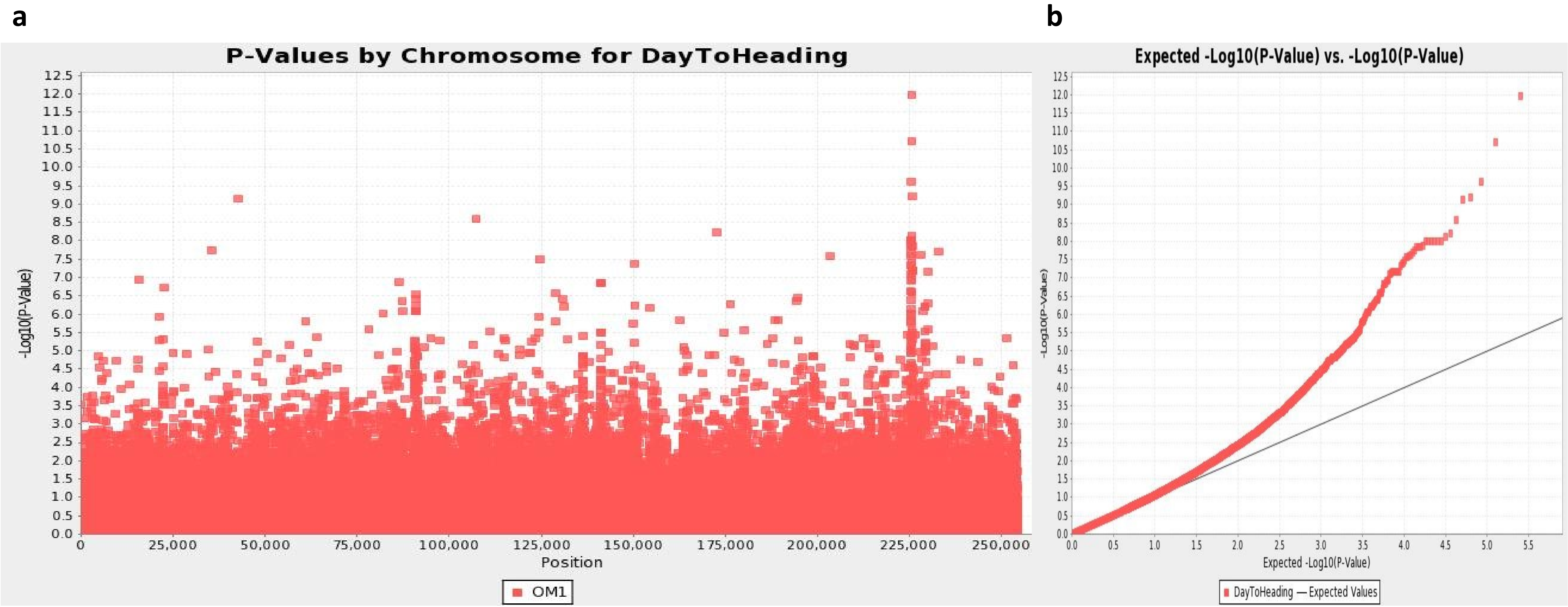

Supplementary Figure S14. The illustration of GWAS result based on the TASSEL analysis using the full 842,474 SNPs resulted kinship matrix and 254,289 synthesized SNPs. The synthesized SNPs are generated from PIP-SNP when configuring the cutoff threshold R_th as 0.6 and choosing "Deep synthesizing by finding the representative Tag SNP. (a.) Manhattan plot. (b.) Q-Q plot

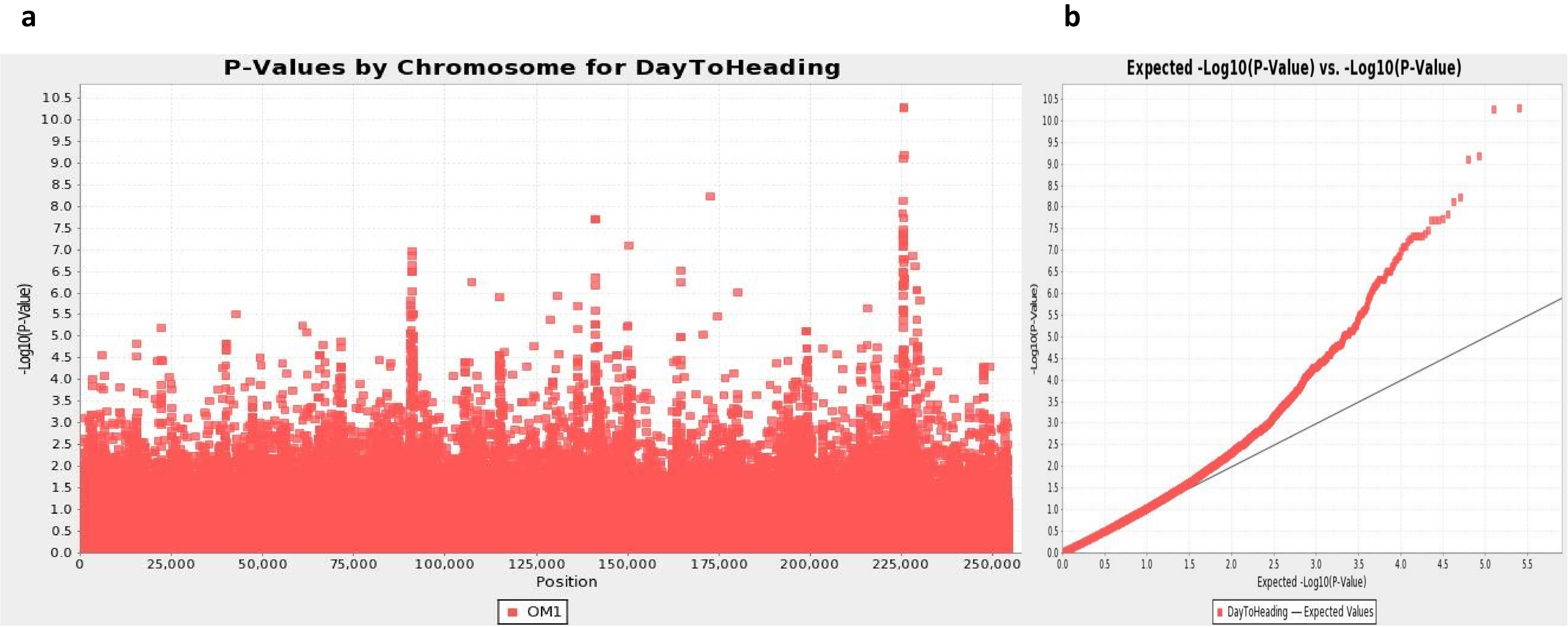

Supplementary Figure S15. The illustration of GWAS result based on the TASSEL analysis using 254,289 synthesized SNPs. The synthesized SNPs are generated from PIP-SNP when configuring the cutoff threshold R_th as 0.6 and choosing “Deep synthesizing by finding the representative Tag SNP. (a.) Manhattan plot. (b.) Q-Q plot

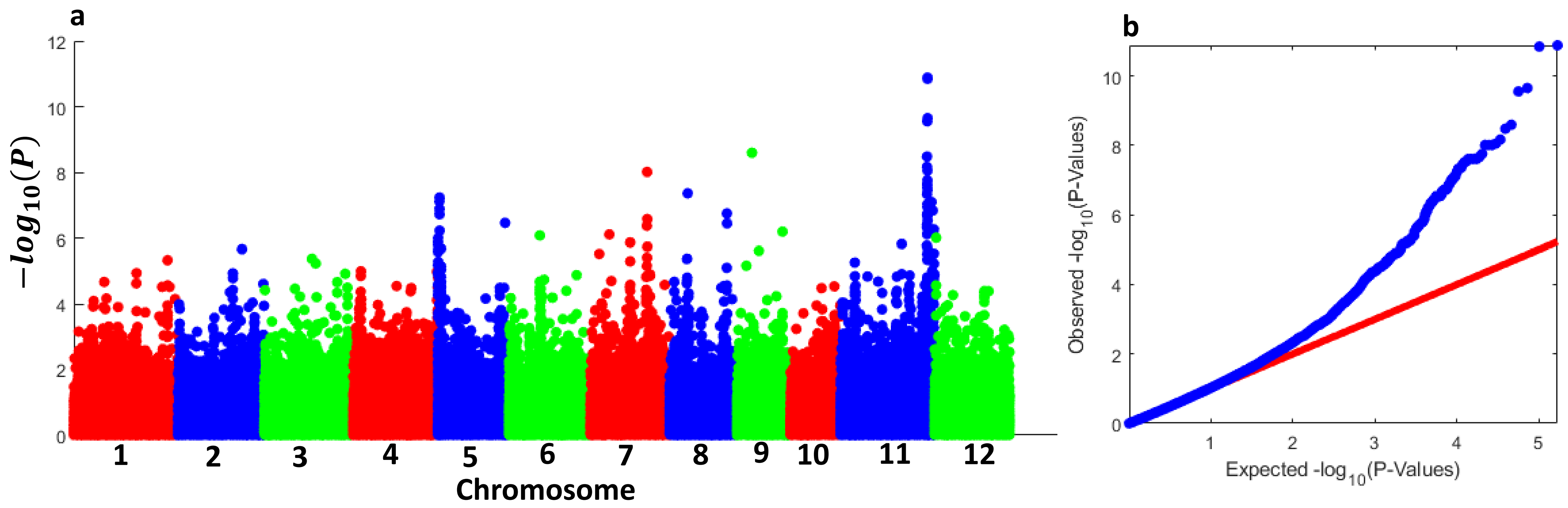

Supplementary Figure S16. The illustration of 1D GWAS result based on the PATOWAS analysis for the 254,289 synthesized SNPs from PIP-SNP when configuring the cutoff threshold R_th as 0.6 and choosing "Deep synthesizing by finding the representative Tag SNP. (a.) Manhattan plot. (b.) Q-Q plot.

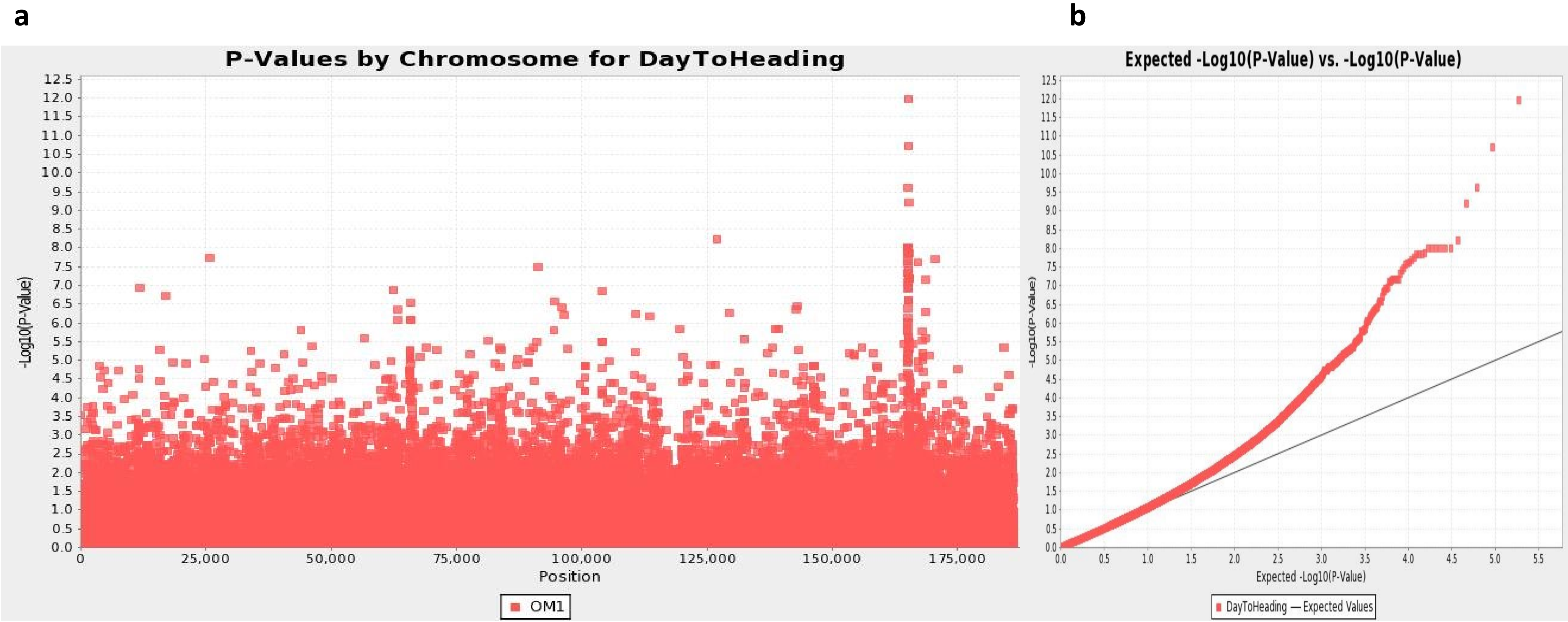

Supplementary Figure S17. The illustration of GWAS result based on the TASSEL analysis using the full 842,474 SNPs resulted kinship matrix and 186,174 synthesized SNPs. The synthesized SNPs are generated from PIP-SNP when configuring the cutoff threshold R_th as 0.4 and choosing "Deep synthesizing by finding the representative Tag SNP. (a.) Manhattan plot. (b.) Q-Q plot

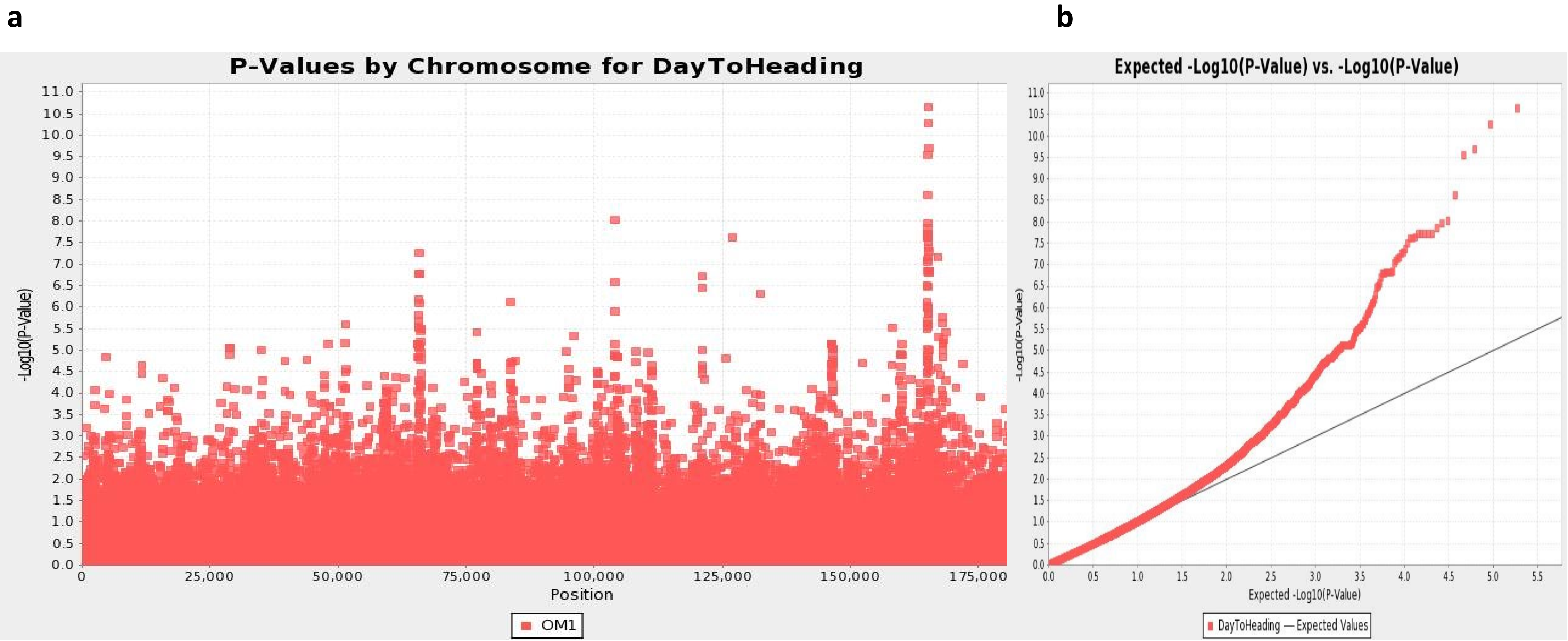

Supplementary Figure S18. The illustration of GWAS result based on the TASSEL analysis using 186,174 synthesized SNPs. The synthesized SNPs are generated from PIP-SNP when configuring the cutoff threshold R_th as 0.4 and choosing “Deep synthesizing by finding the representative Tag SNP. (a.) Manhattan plot. (b.) Q-Q plot

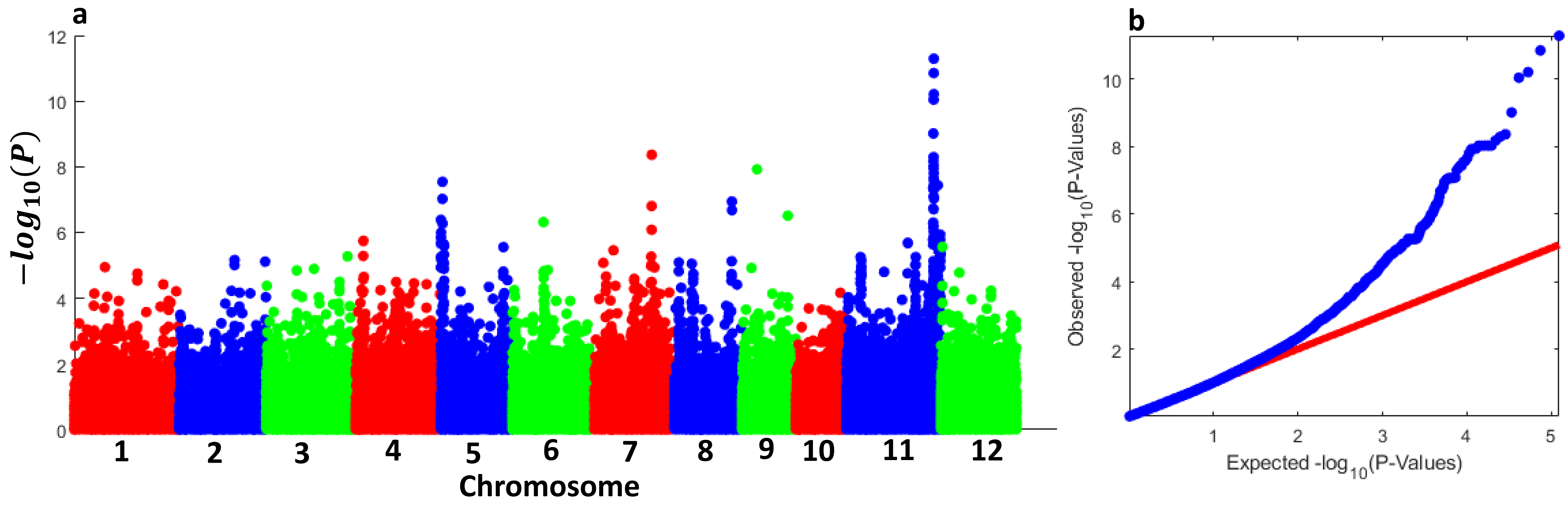

Supplementary Figure S19. The illustration of 1D GWAS result based on the PATOWAS analysis for the 186,174 synthesized SNPs from PIP-SNP when configuring the cutoff threshold R_th as 0.4 and choosing "Deep synthesizing by finding the representative Tag SNP". (a.) Manhattan plot. (b.) Q-Q plot.

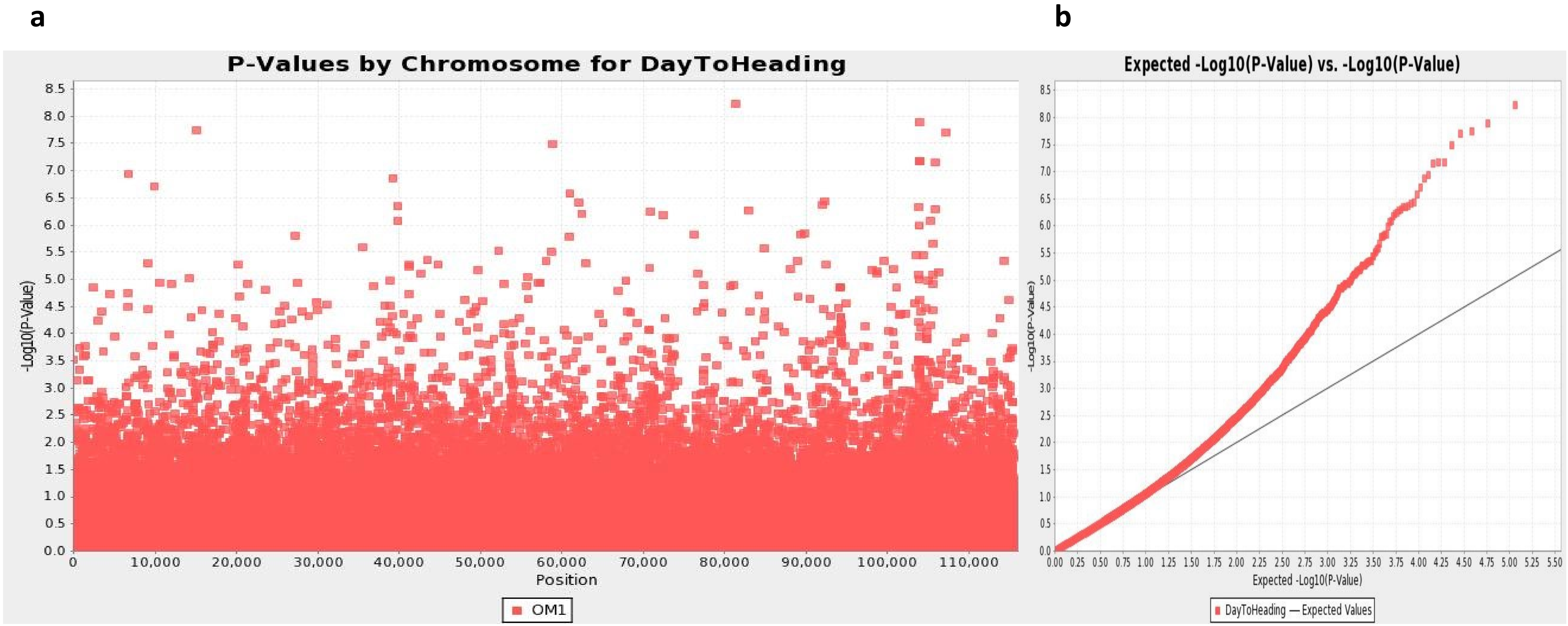

Supplementary Figure S20. The illustration of GWAS result based on the TASSEL analysis using the full 842,474 SNPs resulted kinship matrix and 115,544 synthesized SNPs. The synthesized SNPs are generated from PIP-SNP when configuring the cutoff threshold R_th as 0.2 and choosing “Deep synthesizing by finding the representative Tag SNP. (a.) Manhattan plot. (b.) Q-Q plot

a b

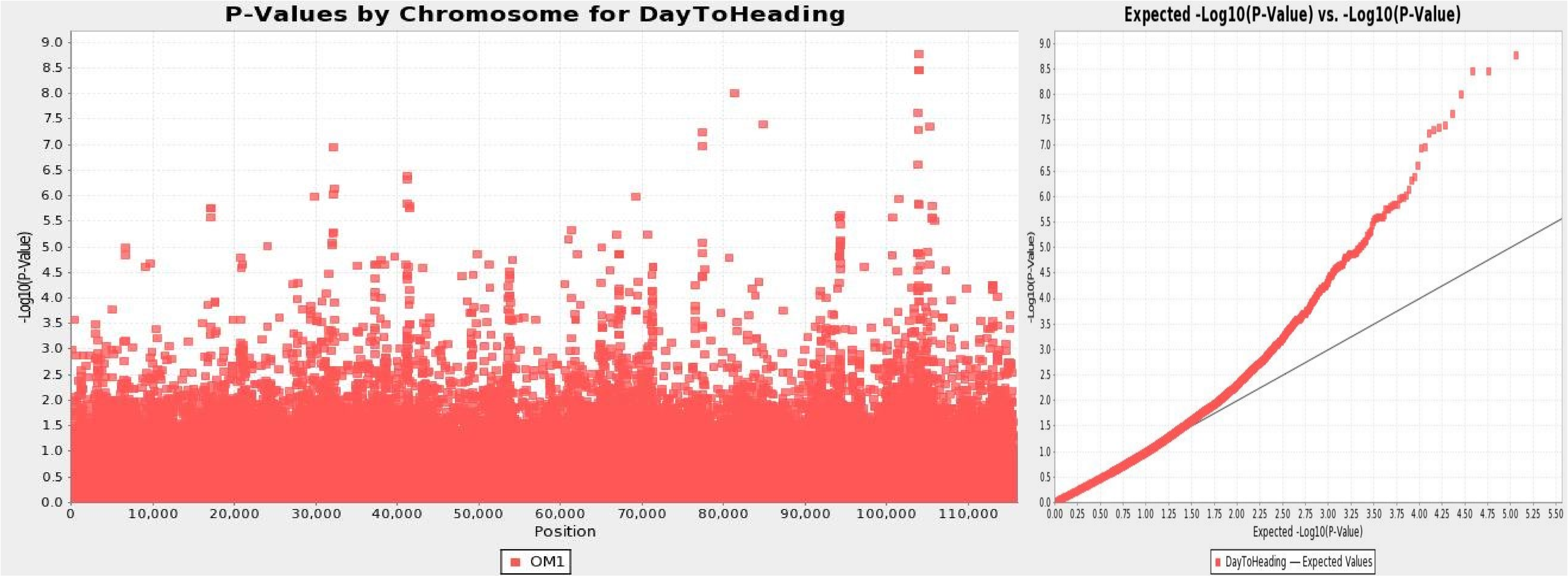

Supplementary Figure S21. The illustration of GWAS result based on the TASSEL analysis using 115,544 synthesized SNPs. The synthesized SNPs are generated from PIP-SNP when configuring the cutoff threshold R_th as 0.2 and choosing "Deep synthesizing by finding the representative Tag SNP. (a.) Manhattan plot. (b.) Q-Q plot

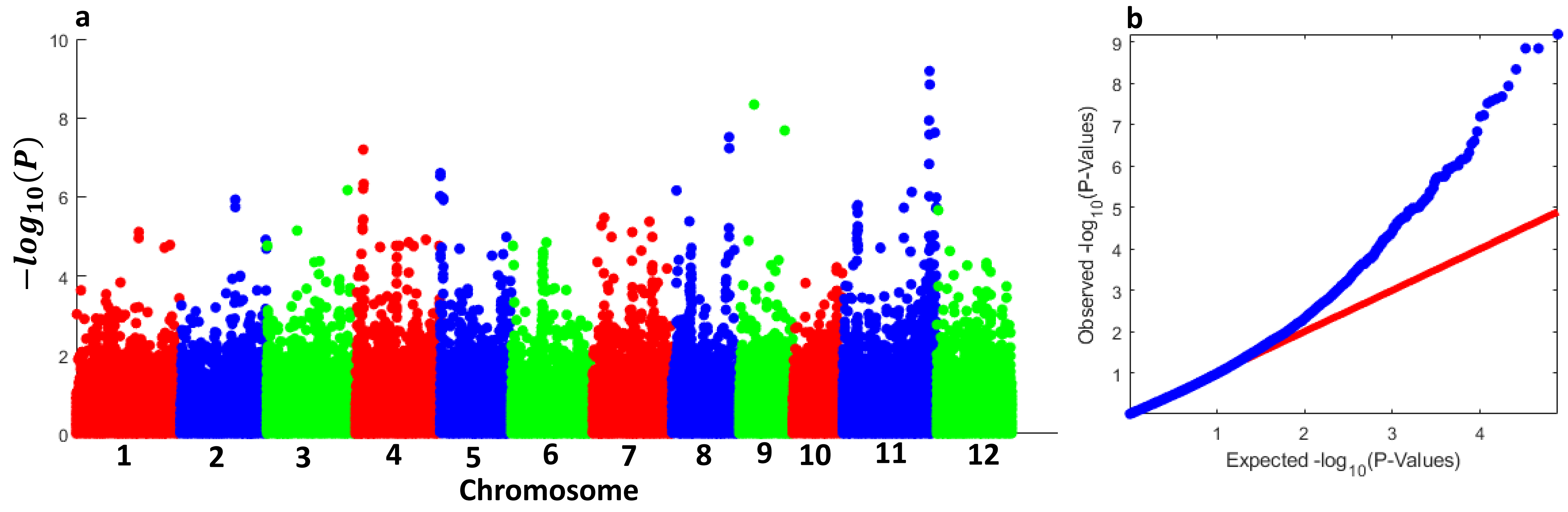

Supplementary Figure S22. The illustration of 1D GWAS result based on the PATOWAS analysis for the 115,544 synthesized SNPs from PIP-SNP when configuring the cutoff threshold R_th as 0.2 and choosing "Deep synthesizing by finding the representative Tag SNP". (a.) Manhattan plot. (b.) Q-Q plot.
