# Supplementary material for: PIP-SNP: a pipeline for processing SNP data featured as linkage disequilibrium bin mapping, genotype imputing and marker synthesizing
Source: NAR Genom Bioinform. 2021 Jul 5;3(3):lqab060. doi: 10.1093/nargab/lqab060 (PMC8256826; doi:10.1093/nargab/lqab060)
Supplement: lqab060_Supplemental_Files [file lqab060_supplemental_files.zip › PIP_SNP_Supplemental_Materials.docx]

# **Supplementary Materials**

PIP-SNP: A Pipeline for Processing SNP Data Featured as Linkage Disequilibrium Bin Mapping, Genotype Imputing and Marker Synthesizing

Wenchao Zhang^1^, Yun Kang^1^, Xinbin Dai^1^, Shizhong Xu^2*^ and Patrick X. Zhao^1*^

^1^Noble Research Institute LLC, 2510 Sam Noble Parkway, Ardmore, OK 73401, USA

^2^Department of Botany and Plant Sciences, University of California, Riverside, CA 92521, USA

Wenchao Zhang, [wezhang@noble.org](mailto:wezhang@noble.org)

Yun Kang, [ykang@noble.org](mailto:ykang@noble.org)

Xinbin Dai, [xdai@noble.org](mailto:xdai@noble.org)

Shizhong Xu, [shizhong.xu@ucr.edu](mailto:shizhong.xu@ucr.edu)

Patrick X. Zhao, [pzhao@noble.org](mailto:pzhao@noble.org)

^*^Corresponding Authors

Shizhong Xu, [shizhong.xu@ucr.edu](mailto:shizhong.xu@ucr.edu), +1-951-827-5898

Patrick X. Zhao, [pzhao@noble.org](mailto:pzhao@noble.org), +1-580-224-6725

**Keywords:** linkage disequilibrium (LD), single-nucleotide polymorphism (SNP) binning, genotype imputation, synthetic marker synthesizing, genome-wide association study (GWAS)

# Supplementary Tables

**Supplementary Table S1.** SNP distribution across the 12 chromosomes in the rice HapMap data

| #Chromosome | 1 | 2 | 3 | 4 | 5 | 6 | 7 | 8 | 9 | 10 | 11 | 12 |
| --- | --- | --- | --- | --- | --- | --- | --- | --- | --- | --- | --- | --- |
| # SNP | 93,822 | 77,730 | 80,116 | 76,183 | 64,108 | 72,941 | 71,378 | 60,724 | 47,948 | 45,289 | 84,665 | 67,570 |

**Supplementary Table S2.** Evaluation result of kinship matrix using the synthesized marker against the original rice HapMap SNP data

| $R\_th$ | 0.8 | 0.6 | 0.4 | 0.2 |
| --- | --- | --- | --- | --- |
| ${RK}_{a}(R\_th)$ | 0.9801 | 0.9637 | 0.9437 | 0.9026 |
| ${RK}_{aa}(R\_th)$ | 0.9810 | 0.9654 | 0.9454 | 0.9037 |
| $RH\left( R\_th \right)$ | 1.0000 | 1.0000 | 1.0000 | 1.0000 |

**Supplementary Table S3.** Function comparison of PIPS-SNP and other tools/platform having related function

|  | SNP Block mapping | Missing value Imputing | Tagging SNP |
| --- | --- | --- | --- |
| PIP-SNP | √ | √ | √ |
| HapBlock (1) | √ |  |  |
| Haploview (2) | √ |  |  |
| LinkImpute (3) |  | √ |  |
| IMPUTE (4) |  | √ |  |
| MaCH (5) |  | √ |  |
| tagsnps.exe (6) |  |  | √ |

# Supplementary Notes

## **Supplementary Note S1.** Description of LD bin detecting and mapping across all SNPs

The SNPs in one LD bin have a relatively high information redundancy, which can be measured by the correlation metrics. Here, we present a method to detect all LD bins to map across all SNPs. One LD bin is characterized by its left boundary and right boundary, while the left boundary is initially fixed, but the right boundary will need to be tentatively tested and finally determined until some criteria are met. To test whether the ${i-1}^{th}$SNP is the right boundary, the criteria can be defined as the comparison between the correlation coefficient of the left and/or right boundary of the bin with the $i^{th}$ SNP against a preset threshold $R\_th$. The $i^{th}$ SNP will not be clustered into the current bin but will be considered as a breakthrough point. **Supplementary** **Figure S1a** illustrated the concept of a LD bin and **Supplementary** **Figure S1b** listed the four options to detect the LD bin’ right boundary. Of all the four method options, the first option is the default method, which can detect the obvious correlation jump as the boundary, but it cannot avoid the scenario when the LD decays gradually. The third option is the most tolerant method, which usually outputs larger LD bin sizes and a smaller number of LD bins, while the forth option is the most critical option, which considers both criteria, and usually outputs more LD bins than options.

Once the right boundary of a bin is determined, the breakthrough point will be considered as the initial left boundary of a new bin. The procedure continues until it finishes mapping of all SNPs. **Supplementary** **Figure S1c** illustrates the flowchart of detecting all LD bins and mapping across all SNPs.

## **Supplementary Note S2.** Description of a modified LD-kNNi method for imputing the missing genotype values in each detected LD bin

Our missing genotypes imputation algorithm was inspired by the LD-kNNi method implemented in the imputation tool LinkImpute (3). We modified LD-kNNi and applied it to each detected LD bin. We describe the modified method as the following.

Suppose a $b\times N$ matrix $G_{b}$ represents a detected LD bin containing $b$ SNP (genotype) markers, the distance of two individual sample $s_{1}$ and $s_{2}$ can be defined by the following equation

$$d_{b}\left( s_{1}{, s}_{2} \right)=\frac{1}{nb} \sum_{p\in P} \left| G_{b}\left( p,s_{1} \right)-G_{b}\left( p,s_{2} \right) \right| (S1)$$

Where $P$ is the set of all possible SNP marker index, and $G_{b}\left( p, s \right)$ is the corresponding genotype values. It is possible that either or both of $G_{b}\left( p,s_{1} \right)$ or $G_{b}\left( p,s_{2} \right)$ are missing, in which case the difference of genotype values should be ignored in the summation. Here, we only consider the cases with two known corresponding genotypes and normalize the summation by known genotype count number $nb$.

Based on the above defined distance between samples, we can use kNN (k-Nearest Neighbor) to impute the missing genotype values in the detected LD bin. For an individual sample $s_{i}$ with missing genotypes to impute, we firstly calculate all the $N-1$ sample pair distance and sort them in ascending order, and then select the top $k$ ‘neighbor’ samples to infer the missing values. The selected $k$ individual samples are defined as sample set *NS*, which may not be connected neighbor samples in the genotype matrix but have the comparatively smaller distance to the specific sample $s_{i}$.

Once the $k$ ‘neighbor’ samples are picked, we can use the following equation to infer the missing genotype $G_{b}\left( p_{j}, s_{i} \right)$

$$G_{b}\left( p_{j}, s_{i} \right)={}_{a\in\{0,1,2\}}^{\arg max}{\sum_{s\in NS} \frac{1}{d_{b}\left( s_{i},s \right)}I\left( G_{b}\left( p_{j},s \right)=a \right)} (S2)$$

Where *NS* is the picked neighbor sample set, $I\left( G_{b}\left( p_{j},s \right)=a \right)$ is the indicator function that takes the value 1 if $G_{b}\left( p_{j},s \right)=a$ and 0 otherwise.

**Supplementary Note S3.** Description of deep synthesizing and options for LD bin marker synthesizing

Compared with the shallow synthesizing, deep synthesizing is an aggressive method. In shallow synthesizing, a LD bin detecting will stop, and its right boundary will be determined as the ${i-1}^{th}$ SNP once the $i^{th}$ SNP meets the preset correlation criteria (**Supplementary** **Figure S1b**). However, in deep synthesizing, a LD bin right boundary detecting will not stop until both the $i^{th}$ and ${i+1}^{th}$SNPs/markers meet the preset correlation criteria. The strategy to test two neighbor SNPs/markers will allow grouping of similar SNPs/markers that are not adjacent to each other. **Supplementary** **Figure S2b** illustrate the whole procedure to implement the module of deep synthesizing. As the bin’s boundary detection proceeds, all the SNPs/markers will be sifted and moved into either a continuous marker buffer or a discontinuous marker buffer, which will be further distinguished into several bins. Here, the continuous marker buffer holds the generally continuous SNPs or markers that are highly correlative and can be synthesized into one marker. All the markers in the continuous marker buffer will be synthesized into one representative marker. The markers in the un-continuous marker buffer originally are neighbor skip marker, but now can be neighbor joint markers (e.g two red continuous markers, two green continuous markers), which can be treated into several bins and further integrated into one synthetic marker per bin. The same procedure in shallow synthesizing can be applied.

Additionally, the input for deep synthesizing is the output from shallow synthesizing (**Supplementary** **Figure S2a**), which can be the integrative markers or the singleton SNPs that are not correlated to their left and right neighbor SNPs but may be correlated to their skip neighbor SNPs.

To a detected LD bin, a synthetic marker that represents the bin can be generated by two methods. Considering the two choices as shallow and deep synthesizing, PIP-SNP provides five options for LD bin marker synthesizing: imputing only with no synthesizing, shallow synthesizing by integrating all SNPs, shallow synthesizing by finding the tag SNPs, deep synthesizing by integrating all SNPs, and deep synthesizing by finding the tag SNPs. Shallow synthesizing is limited in reducing the SNP marker dimension, while the deep synthesizing method can achieve that, especially for random Hapmap SNP data. The integration method has a better performance in conserving the genetic information, but the marker resolution will be degraded into bins, while the tag SNP selection method will keep the original SNP format.

**Supplementary Note S4.** The mathematical bases that the integration method conserves more genetic information in the delivered kinship matrix

In this note, we will provide the mathematical bases of the integration method to generate the synthesis marker and its performance in conserving genetic information in the resulted kinship matrix [7-9]. Let $G$ be a SNP-derived genotype matrix as dimension of $M\times N$, where $M$ is the marker number and $N$ is the individual number. Suppose$b$ SNP (genotype) markers are detected as a LD block/bin (discriminated as the bold part).

$G=\left[ \begin{matrix} \begin{matrix} \boldsymbol{g}_{\boldsymbol{11}} & \boldsymbol{g}_{\boldsymbol{12}} \\ \boldsymbol{g}_{\boldsymbol{21}} & \boldsymbol{g}_{\boldsymbol{22}} \\ \begin{matrix} \boldsymbol{\ldots} \\ \boldsymbol{g}_{\boldsymbol{b}\boldsymbol{1}} \end{matrix} & \begin{matrix} \boldsymbol{\ldots} \\ \boldsymbol{g}_{\boldsymbol{b}\boldsymbol{2}} \end{matrix} \end{matrix} & \cdots& \begin{matrix} \boldsymbol{g}_{\boldsymbol{1}\boldsymbol{N}} \\ \boldsymbol{g}_{\boldsymbol{2}\boldsymbol{N}} \\ \begin{matrix} \boldsymbol{\ldots} \\ \boldsymbol{g}_{\boldsymbol{bN}} \end{matrix} \end{matrix} \\ \vdots& \ldots& \vdots\\ \begin{matrix} g_{M1} & g_{M2} \end{matrix} & \cdots& g_{MN} \end{matrix} \right]$ (S3)

Therefore, the $G$ matrix can be considered as two parts: markers inside the LD bin and other markers outside the LD bin, which are represented as $G_{b}$ and $G_{\bar{b}}$ , respectively,

$G=\left[ \begin{matrix} G_{b} \\ G_{\bar{b}} \end{matrix} \right]$ (S4)

As we know, the marker-based kinship matrix (Astle and Balding, 2009; Bernardo, et al., 1996; Zhang, et al., 2019), $K$ is a matrix with dimension of $N\times N$ and follow the property of linearity.

$K=G^{'}*G=K_{b}+K_{\bar{b}}=G_{b}^{'}*G_{b}+G_{\bar{b}}'*G_{\bar{b}}$ (S5)

The $b$ SNP markers in the detected LD block will be synthesized into one representative marker. Mathematically, there are many methods to synthesize. We want to keep the integrity of the original kinship matrix as much as possible. Here, we use Euclidean norm2 to synthesize the$b$ markers into one marker, which is represented as

$G_{bs}=\left[ \begin{matrix} \sqrt{\sum_{i=1}^{b} {g_{i1}}^{2}} & \sqrt{\sum_{i=1}^{b} {g_{i2}}^{2}} & \begin{matrix} \ldots& \sqrt{\sum_{i=1}^{b} {g_{N1}}^{2}} \end{matrix} \end{matrix} \right]$ (S6)

$G_{bs}$ is vector as dimension of $1\times N$

Using only the synthesized marker $G_{bs}$, we can calculate a partial kinship matrix as

$K_{bs}=G_{bs}^{'}*G_{bs}$ (S7)

because

$G_{b}=\left[ S_{b}\left( 1 \right); S_{b}\left( 2 \right);\ldots S_{b}\left( i \right);\ldots; S_{b}\left( N \right) \right]$ (S8)

$S_{b}\left( i \right)$ corresponds to a column vector containing $b$ genotype values for individual $i$, and its Euclidean norm can be represented as $Norm\left( S_{b}\left( i \right) \right)=\left\| S_{b}\left( i \right) \right\|_{2}$

$\left\| S_{b}\left( i \right) \right\|_{2}=\sqrt{\sum_{i=1}^{b} {g_{i1}}^{2}}$ (S9)

Define a correlation coefficient between individual $i$ and $j$ as

$R_{b}\left( i,j \right)=\frac{\sum_{k=1}^{b} S_{b}\left( i \right)^{\circ}S_{b}\left( j \right)}{\left\| S_{b}\left( i \right) \right\|_{2}\left\| S_{b}\left( j \right) \right\|_{2}}$ (S10)

where $S_{b}\left( i \right)^{\circ}S_{b}\left( j \right)$ represents the dot product of two vectors of $S_{b}\left( i \right)$ and $S_{b}\left( j \right)$, and results in a column vector containing $b$ items.

Obviously, for the original SNP marker block, the resulted block kinship matrix is:

$K_{b}=\left[ \begin{matrix} \sum_{k=1}^{b} S_{b}\left( 1 \right)^{\circ}S_{b}\left( 1 \right) & \cdots& \sum_{k=1}^{b} S_{b}\left( n \right)^{\circ}S_{b}\left( 1 \right) \\ \vdots& \ddots& \vdots\\ \sum_{k=1}^{b} S_{b}\left( 1 \right)^{\circ}S_{b}\left( N \right) & \cdots& \sum_{k=1}^{b} S_{b}\left( N \right)^{\circ}S_{b}\left( N \right) \end{matrix} \right]$ and $K_{b}\left( i,j \right)=\sum_{k=1}^{b} S_{b}\left( i \right)^{\circ}S_{b}\left( j \right)$ (S11)

While for the synthesized markers, the resulted block kinship matrix is:

$K_{bs}=\left[ \begin{matrix} \left\| S_{b}\left( 1 \right) \right\|_{2}\left\| S_{b}\left( 1 \right) \right\|_{2} & \cdots& \left\| S_{b}\left( N \right) \right\|_{2}\left\| S_{b}\left( 1 \right) \right\|_{2} \\ \vdots& \ddots& \vdots\\ \left\| S_{b}\left( 1 \right) \right\|_{2}\left\| S_{b}\left( N \right) \right\|_{2} & \cdots& \left\| S_{b}\left( N \right) \right\|_{2}\left\| S_{b}\left( N \right) \right\|_{2} \end{matrix} \right]$ and $K_{bs}\left( i,j \right)=\left\| S_{b}\left( i \right) \right\|_{2}\left\| S_{b}\left( j \right) \right\|_{2}$ (S12)

From S8-10, there is a mathematical relationship as

$K_{bs}\left( i,j \right)=\frac{K_{b}\left( i,j \right)}{R_{b}\left( i,j \right)}$ (S13)

because $R_{b}\left( i,j \right)\leq1.0$ therefore $K_{bs}\left( i,j \right)\geq K_{b}\left( i,j \right)$.

It’s worth to note that the diagonal elements of $K_{b}$ and $K_{bs}$ are always equal, because $R_{b}\left( i,i \right)=1.0$, which further ensures that $K_{b}\left( i,i \right)=K_{bs}\left( i,i \right)$.

Additionally, if the detected $b$ SNP markers are from a LD block composed of tightly correlated SNPs (having much higher Pearson $R^{2}$ values or D values that are close to 1.0), the individual $S_{b}\left( i \right)$ and $S_{b}\left( j \right)$ have the close evolution relationship as $S_{b}\left( i \right)\approx S_{b}\left( j \right)$, therefore, $R_{b}\left( i,j \right)$ are also very close to 1.0. In the ideal case, all the markers in one LD block are identical markers, $S_{b}\left( i \right)==S_{b}\left( j \right)$, $R_{b}\left( i,j \right)==1.0$, and $K_{b}=K_{bs}$

In summary, if we choose the Euclidean norm to generate synthesis markers representing the LD blocks composed of tightly correlated SNPs, the kinship matrix generated using the synthesized markers will deviate minimally from its peer using the original SNP markers.

# References

1. Zhang, K., Deng, M., Chen, T., Waterman, M.S., and Sun, F. (2002) A dynamic programming algorithm for haplotype block partitioning. *PNAS*, 99:7335-7339.

2. Barrett, J.C., Fry, B., Mailer, J., and Daly, M.J. (2005) Haploview: analysis and visualization of LD and haplotype maps. *Bioinformatics,*21:263-265.

3. Money, D., Gardner, K., Migicovsky, Z., Schwaninger, H., Zhong, G.Y. and Myles, S. (2015) LinkImpute: Fast and Accurate Genotype Imputation for Nonmodel Organisms. *G3 (Bethesda)*, 5, 2383-2390.

4. Howie, B. N., Donnelly, P., and Marchini, J. (2009) A flexible and accurate genotype imputation method for the next generation of genome-wide association studies. *PLoS Genetics* 5(6): e1000529.

5. Li, Y., Willer, C.J., Ding, J., Scheet, P., and Abecasis, G.R. (2010) MaCH: using sequence and genotype data to estimate haplotypes and unobserved genotypes. *Genet Epidemiol*, 34(8):816-34.

6. Stram, D.O., Haiman, C.A., Hirschhorn, J.N., Altshuler, D., Kolonel, L.N., Henderson, B.E., and Pike, M.C. (2003) Choosing haplotype tagging SNPs based on unphased genotype data using a preliminary sample of unrelated subjects with an example from the multiethnic cohort study. *Hum. Hered*. 55:27–36.

7. Astle, W. and Balding, D.J. (2009) Population Structure and Cryptic Relatedness in Genetic Association Studies, *STATISTICAL SCIENCE*, 24:451 - 471.

8. Bernardo, R., Murigneux, A. and Karaman, Z. (1996) Marker-based estimates of identity by descent and alikeness in state among maize inbreds. *Theoretical and Applied Genetics*, 93(1):262-267.

9. Zhang, W., Dai, X., Xu, S., and Zhao P.X*.* (2019) GPU empowered pipelines for calculating genome-wide kinship matrices with ultra-high dimensional genetic variants and facilitating 1D and 2D GWAS. *NAR Genomics and Bioinformatics,* 2(1).
